# Promoting the transition from pyroptosis to apoptosis in endothelial cells: a novel approach to alleviate methylglyoxal-induced vascular damage

**DOI:** 10.1186/s12967-025-06195-x

**Published:** 2025-02-10

**Authors:** Ruqiang Yuan, Hu Xu, Mingqi Wang, Lina Guo, Yang Yao, Xiaoru Zhang, Xiuli Wang

**Affiliations:** 1 College of Basic Medical Science, Dalian Medical University, Dalian, 116044 China; 2 Advanced Institute for Medical Sciences, Dalian Medical University, Dalian, 116044 China; 3 Health Science Center, East China Normal University, Shanghai, 200241 China

**Keywords:** Methylglyoxal, Vascular endothelial cells, Pyroptosis-apoptosis transition, Vascular organoid, DT-13

## Abstract

**Background:**

Methylglyoxal (MGO)-induced cell death in vascular endothelial cells (VECs) plays a critical role in the progression of diabetic vascular complications (DVCs). Previous studies have shown that MGO can induce inflammatory pyroptosis, leading to VEC damage. However, the underlying mechanism remains unclear, and effective interventions are yet to be developed.

**Methods:**

Human umbilical vein endothelial cells (HUVECs) were used for in vitro experiments. Cell death modes were assessed through morphological observations. Mechanistic investigations were performed using immunofluorescence, flow cytometry, Western blotting, and ELISA. Inhibitors and adenoviruses were employed to validate the mechanisms. Vascular organoids in conjunction with AngioTool plug-in assays were used to evaluate VEC damage and angiogenic capacity. Mouse blood pressure was measured using the tail-cuff method, and vascular morphology was examined through hematoxylin and eosin (H&E) staining as well as immunofluorescence staining. Data were analyzed using the GraphPad Prism software.

**Results:**

Our study revealed that MGO induces pyroptosis in VECs via the Caspase3/gasdermin E (GSDME) pathway. Furthermore, the saponin monomer 13 of dwarf lilyturf tuber (DT-13), inhibited MGO-induced pyroptosis and promoted the generation of apoptotic bodies, facilitating the transition from pyroptosis to apoptosis. Mechanistically, DT-13 suppressed the Caspase3-mediated cleavage of GSDME and non-muscle myosin heavy chain IIA (NMMHC IIA), while increasing the phosphorylation of myosin light chain 2 (MLC2), which facilitated apoptotic body formation. Additionally, DT-13 was shown to mitigate VEC damage, inhibit angiogenesis, reduce vascular remodeling, and alleviate MGO-induced hypertension.

**Conclusions:**

This study uncovers a novel mechanism through which MGO induces VEC damage, highlighting the therapeutic significance of the transition from pyroptosis to apoptosis in this process. These findings suggest potential therapeutic strategies for managing diabetic angiopathy. Furthermore, DT-13 emerges as a promising compound for therapeutic intervention, offering new possibilities for clinical applications.

**Supplementary Information:**

The online version contains supplementary material available at 10.1186/s12967-025-06195-x.

## Introduction

Cell death is a fundamental process through which organisms respond to external stimuli and maintain homeostasis [1]. Historically, cell death pathways were considered distinct, with minimal overlap [2]. For example, pyroptosis and apoptosis—both forms of programmed cell death (PCD) mediated by caspase proteins—were once thought to operate independently [1, 3, 4]. Apoptosis is primarily triggered by death receptors (e.g., DR3/4/5, Fas, TNFR1/2) [5], mitochondrial outer membrane permeabilization (MOMP) [6, 7], the translocation of Bad/Bax proteins to the outer mitochondrial membrane [8], and the release of apoptotic protease activating factor-1 (Apaf-1) [9, 10], cytochrome c [11, 12], and dATP [9], leading to the activation of Caspase8/9, which in turn activates Caspase3/7. The activated Caspase3/7 then trigger a cascade of reactions that execute apoptosis [13, 14]. In contrast, pyroptosis is triggered through both classical and non-classical pathways that activate Caspase1/4/5/11, resulting in the cleavage of gasdermin D (GSDMD), and the generation of N-terminal fragments that target the plasma membrane to form pores, which induces pyroptosis [15, 16]. Thus, it was previously believed that Caspase3 activation and GSDMD cleavage were unique features of apoptosis and pyroptosis, respectively [17, 18].

However, recent studies have shown that GSDME, another member of the gasdermin family, can be cleaved by Caspase3 at its Asp270 position, generating N-terminal fragments that trigger pyroptosis [19]. This discovery challenges the notion that pyroptosis and apoptosis are independent processes. Consequently, the concept of a “Caspase3/GSDME signaling pathway” has emerged as a switch for transitioning between apoptosis and pyroptosis, revealing interconnected signaling pathways between these two forms of cell death [17, 18]. Modulating key molecules within these pathways can induce a switch in the mode of cell death. A growing body of literature supports this idea. For instance, studies suggest that the transition from apoptosis to pyroptosis can be therapeutically exploited in cancer treatment. High expression of GSDME enables traditional chemotherapy agents like Cisplatin and Paclitaxel to activate the Caspase3/GSDME pathway and induce pyroptotic cell death [20]. Additionally, compounds like the piperlongumine analogue L50377 enhances this process by increasing reactive oxygen species (ROS) [21]. This transition has also been demonstrated in various cancer models, including melanoma, osteosarcoma, and gastrointestinal cancers [18], highlighting the potential for improving treatment efficacy by modulating cell death pathways. However, further exploration is needed to identify specific mechanisms and potential interventions in other diseases, such as cardiovascular diseases. This is particularly important, as clarifying these mechanisms would contribute significantly to achieving effective, mechanism-based treatment outcomes for cardiovascular diseases.

DVCs are major causes of morbidity and mortality in diabetic patients [22]. Recent research has identified MGO, a non-enzymatic byproduct of glucose metabolism, as a key factor in the progression of DVCs [23–26]. MGO accumulates pathologically in VECs and triggers a series of pathological processes, including oxidative stress, inflammation, and cell death [27–30]. Studies suggest that MGO induces various modes of cell death in VECs, including apoptosis via excessive mitochondrial ROS accumulation and dysregulation of endothelial nitric oxide synthase (eNOS) [31, 32], as well as pyroptosis via the NLRP3 inflammasome [33]. These findings underscore the complexity of MGO-induced cell death in VECs. However, the intrinsic connections and shared mechanisms between MGO-induced pyroptosis and apoptosis, as well as the pathways governing their conversion remain unclear, and effective therapeutic strategies are still lacking. This study aims to address this gap by investigating the molecular mechanisms underlying MGO-induced pyroptosis in VECs through in vitro experiments and identifying potential therapeutic agents that could promote the transition from pyroptosis to apoptosis.

We employed HUVECs as a model system, using a combination of morphological observations, Western blotting, flow cytometry, and immunofluorescence techniques to systematically study the effects of MGO on VEC pyroptosis and the associated signaling pathways. Our findings demonstrate that MGO induces GSDME-dependent pyroptosis in HUVECs. Based on this, we screened the natural compound DT-13, which effectively inhibits MGO-induced pyroptosis in HUVECs and promotes the conversion of pyroptotic cells to apoptotic ones. Furthermore, we explored the regulatory effects of DT-13 on the pyroptosis-apoptosis transition (PAT) and its underlying molecular mechanisms, revealing its association with Rho-associated coiled-coil containing protein kinase (ROCK)-mediated MLC2 phosphorylation and NMMHC IIA protein regulation. Finally, the DT-13 protective effects on VECs and blood vessels were confirmed using vascular organoids and mouse models. Thus, this study investigates potential intervention strategies against MGO-induced endothelial cell damage by switching cell death modes, contributing to a better understanding of DVC pathogenesis and offering new insights for treatment strategies.

## Methods

### Cell culture and treatment

HUVECs and human embryonic stem cell line H9 (hESCs) were obtained from the ATCC. HUVECs were cultured in ECM (#1001, Sciencell, USA) supplemented with 5% fetal bovine serum (FBS), 100 U/mL penicillin, and 100 µg/mL streptomycin. hESCs (H9) were cultured in mTeSRTM Plus Basal Medium (100–0274, STEMCELL, CA) with mTeSRTTM Plus 5 × Supplement (100–0275, STEMCELL) in Matrigel (354234, Corning, USA)-coated 6-well plates. Cells were passaged when they reached 70–80% confluence, and the medium was replenished every two days.

In the experiment to investigate the effects of MGO (M0252, Sigma, USA), various inhibitors, including N-Acetylcysteine (NAC, 10 µM, S1623, Selleck, USA), Z-VAD-FMK (30 µM, S7023, Selleck), and Z-DEVD-FMK (30 µM, S7312, Selleck), were administered 2 h prior to MGO treatment. DT-13 (1 µM, 130551-41-6, MCE, China) was administered 6 h prior to MGO. Additionally, Y27632 (5 µM, Y0503, Sigma) and Blebbistatin (Bleb, 5 µM, HY-13441, MCE) were added 2 h before DT-13 treatment to evaluate their effects on the PAT. Furthermore, calcium-free medium (PWL034, Meilunbio, China) and 1 mM Ca²⁺ (CaCl_2_, 449709, Sigma) were used to explore the effect of Ca²⁺ on DT-13.

### CCK-8 assay

To establish the optimal dosages of MGO and DT-13, HUVECs were seeded in 96-well plates at a density of 1 × 10^4^ cells per well to reach approximately 70% confluence. After adherence, the cells were treated with FBS-free medium for 6 h. Subsequently, different concentration MGO (0, 0.2, 0.4, 0.8 and 1.6 mM) and DT-13 (0, 0.01, 0.1, 1, and 10 µM) were added to the medium followed by additional 24 h of incubation. Finally, a microplate reader (TECAN Spark, Austria) was used to measure the absorbance at 450 nm after adding CCK-8 reagent solution according to the manufacturer’s instructions to calculate cell viability.

### Crystal violet stain and ROS detection

HUVECs were adhered to 6-well plates and treated in serum-free medium for 6 h, followed by treatment with medium containing different concentrations of MGO (MGO: 0, 0.2, 0.4, 0.8 and 1.6 mM) for 24 h and photographed. Cell viability was assessed via crystal violet staining, and images were captured using a digital camera (Canon70D, Japan). To assess ROS production, cells were incubated with 10 µM 2’,7’-dichlorodihydrofluorescein diacetate (DCFH-DA) (S0033M, Beyotime) in serum-free medium at 37 °C for 20 min. ROS levels were visualized and captured using a fluorescence microscope (Leica DM4B, Germany), with excitation and emission wavelengths of 488 nm and 525 nm.

### Flow cytometry analysis

Cell samples for flow cytometry were collected via centrifugation and gently resuspended in 100 µL of staining buffer (E-CK-A107, Elabscience, China). The cells were then stained with 5 µL of Annexin V-FITC solution (E-CK-A111, Elabscience) and 10 µL of propidium iodide (PI, E-CK-A161, Elabscience). After a 20-minute incubation in the dark at room temperature, 400 µL of additional staining buffer was added. Samples were analyzed using a BD Aria III flow cytometer (USA).

### Western blotting

Samples were harvested and lysed in lysis buffer, with 15 µg of protein was used for analysis. Protein samples were separated by 10-12% SDS-PAGE and subsequently transferred to a PVDF membrane (BSP0161, PALL, USA). The membrane was incubated with the primary antibodies listed below: rabbit anti-GSDME (ab215191, Abcam), rabbit anti-VE-Cadherin (ab33168, Abcam), rabbit anti-Caspase3 (9662 S, CST, USA), rabbit anti-MLC2 (3672 S, CST), rabbit anti-phospho-myosin light chain 2(p-MLC2) (3671 S, CST), rabbit anti-PARP (ET608-56, HuaBio, China), rabbit anti-Cleaved-PARP (ET1608-10, HuaBio), and rabbit anti-Myosin heavy chain 9 (MYH9) (11128-1-AP, Proteintech). After washing, the membrane was incubated with horseradish peroxidase-conjugated secondary antibodies, and the immunoreactivity was detected using enhanced chemiluminescence (SQ201, Epizyme, China). Anti-β-actin (ab8226, Abcam) was used as a loading control to verify equal protein loading.

### LDH and IL-18 measurement

The supernatant of the cell culture was collected to measure the lactate dehydrogenase (LDH) content using an LDH assay kit (A020-2-2, Njjcbio, China). Additionally, the supernatant was used to measure the content of interleukin 18 (IL-18) using an IL-18 ELISA kit (E-EL-H0253, Elabscience). Following the manufacturer’s protocols, the optical density (OD) values of LDH and IL-18 were detected using a microplate reader (TECAN Spark, Austria) at wavelengths of 440 nm and 450 nm, and the concentrations were calculated.

### Morphological observation of apoptotic bodies

HUVECs were cultured in 6-well plates and subjected to various treatments to induce apoptosis. After treatment, morphological changes were observed, and recorded using an inverted microscope (Olympus CKX53, Japan). Images were captured to assess the effects of the treatments on cellular morphology, focusing particular on cell shrinkage, membrane blebbing, and the formation of apoptotic bodies.

### MYH9 adenovirus infection

HUVECs were cultured in 10 cm diameter dishes at a cell density of approximately 30%. After the cells had adhered, a multiplicity of infection (MOI) of 150 for both MYH9 overexpressing adenovirus and negative control adenovirus (both obtained from Vigene Biosciences, China) was added, respectively. After a 12-hour infection, the medium was replaced with fresh medium containing 2% FBS, and the cells were cultured for an additional 72 h. The cells were then digested and seeded onto 6-well plates at a density of approximately 70%. After adhering, the cells were subjected to various treatments and subsequently observed and photographed.

### Construction of hVOs and morphological analysis

Human vascular organoids (hVOs) were constructed according to methods described in previous literature [34]. Firstly, hESCs (H9) were aggregated in suspension culture. To induce mesodermal differentiation, the medium consisted of 50% Neurobasal medium and 50% DMEM/F12, supplemented with 0.5% Glutamax, 2% B27, 1% N2 supplement, 1% P/S (all from Gibco), 0.143 mM β-mercaptoethanol (β-me, 516732, Sigma), 30 ng/mL BMP4 (HZ-1045, Proteintech, China), and 12 µM CHIR99021 (A3011, APExBIO, USA). After 3 days of differentiation, the medium was replaced with vascular lineage induction medium, comprising 50% Neurobasal medium, 50% DMEM/F12, 0.5% Glutamax, 2% B27, 1% N2 supplement, 1% P/S, 0.143 mM β-me, 100 ng/mL VEGFA (HZ-1038, Proteintech), and 2 µM Forskolin (F3917, Sigma). On day 8, the medium was changed to StemPro^®^-34 SFM medium (10640-019, Gibco) supplemented with 1% Glutamax, 1% P/S, 15% FBS, 100 ng/mL FGF-2 (100-18B-100, Peprotech, USA), and 100 ng/mL VEGFA. From day 10 onwards, cell aggregates were embedded in a composite hydrogel (collagen: Matrigel = 3:1, v/v) and cultured in the vascular network promotion medium, with medium changes every 3 days thereafter. After 5 days of incubation, the hVOs were ready for characterization and subsequent application.

The images of hVOs were captured using an inverted microscope (Olympus CKX53, Japan) and then binarized with ImageJ software (1.53 K, NIH) The binarized vascular networks were subsequently analyzed using the AngioTool plug-in. Statistical analysis of parameters, including the number of nodes, junctions, segments, branches, and total lengths of segments and branchs, was performed using Prism software (10.2.3, GraphPad Software, Inc).

### Blood pressure monitoring in mice

Blood pressure of 8-week-old male C57BL/6 mice was measured with the tail-cuff method (Softron BP2010, China). To minimize fluctuations related to stress, blood pressure was measured daily for one week prior to the experiment to allow the mice to acclimate to their environment. The mice were divided into four groups: Control, MGO model, DT-13 treatment (5 mg/kg), and DT-13 treatment (10 mg/kg). The treatment protocols were as follows:

A. Intraperitoneal Injection: Mice in the DT-13 treatment and MGO model groups received an intraperitoneal injection of 50 mg/kg MGO (10 µg/µL) daily, while the control mice received saline daily.
B. Oral Gavage: Mice in the DT-13 treatment groups received 5 mg/kg or 10 mg/kg DT-13 (1 µg/µL), while the control and MGO model groups received the corresponding vehicle solution (0.5% CMC-Na solution containing 0.1% DMSO) daily.

The duration of the experiment was 3 weeks, during which blood pressure was measured and recorded weekly. All mice were obtained from the Animal Experiment Center of Dalian Medical University, and the animal experimentation protocol was approved by the Animal Ethics Committee of Dalian Medical University.

### H&E and immunofluorescence staining

Samples were fixed in 4% paraformaldehyde solution (G1101, Servicebio, China) for 24 h. After being dehydrated in 20% sucrose solution for over 48 h, the mesentery was embedded in optimal cutting temperature compound (OCT) and sectioned to a thickness of 5 μm. Sections were stored at -20 °C. For H&E staining, sections were rinsed with phosphate-buffered saline (PBS), stained with hematoxylin dye for 2 min, differentiated using 1% hydrochloric acid, and then washed with distilled water. Eosin dye was then applied, and excess dye was removed. The samples were subsequently dehydrated, sealed, and observed under a microscope.

For immunofluorescence staining, tissue sections or fixed cells were permeabilized with 0.1% Triton X-100 and subsequently blocked with 5% BSA at room temperature for 1 h. The samples were then incubated overnight at 4 °C with primary antibodies against MYH9 (11128-1-AP, Proteintech), α-SMA (Mouse monoclonal, A5228, Sigma), and CD31 (Rabbit monoclonal, ab182981, Abcam). Following this, samples were incubated with Alexa Fluor^®^ 488-labeled goat anti-rabbit and mouse IgG (ZF-0511/ZF-0512, ZSGB-BIO, China) and Alexa Fluor^®^ 594-labeled goat anti-rabbit IgG (ZF-0516, ZSGB-BIO).

For cytoskeletal staining, samples were incubated in the dark with Rhodamine Phalloidin (C2205S, Beyotime, China) diluted 1:200 at room temperature for 1 h. Nuclei were counterstained with DAPI (C0060, Solarbio) at room temperature for 20 to 30 min.

For whole-mount immunostaining of hVOs, all procedures adhered to the standard immunofluorescence protocol described above, with extended incubation times, specifically, 4 h for permeabilization and 2 h for blocking. After blocking, samples were incubated with Alexa Fluor^®^ 647-conjugated CD31 antibody (558094, BD Biosciences, USA) at 4 °C for 16 to 20 h in the dark. Following incubation, organoids were washed twice with PBS, and nuclei were counterstained with DAPI for 2 h. Fluorescent images were captured, processed, and saved using a laser confocal microscope (SP8, Leica).

### Statistical analysis

The data are expressed as the mean ± SD, and statistical analysis was performed using Student’s t-test for comparisons between two groups. Normality was assessed with the D’Agostino-Pearson omnibus test, and homogeneity of variances was evaluated with the Brown-Forsythe test. Differences among multiple groups were analyzed using one-way or two-way ANOVA, followed by appropriate post-hoc tests. A significance level of *P* < 0.05 was considered statistically significant. All experiments were conducted in triplicate unless otherwise specified.

## Results

### MGO reduces HUVEC viability and induces cell death

**Fig. 1:**
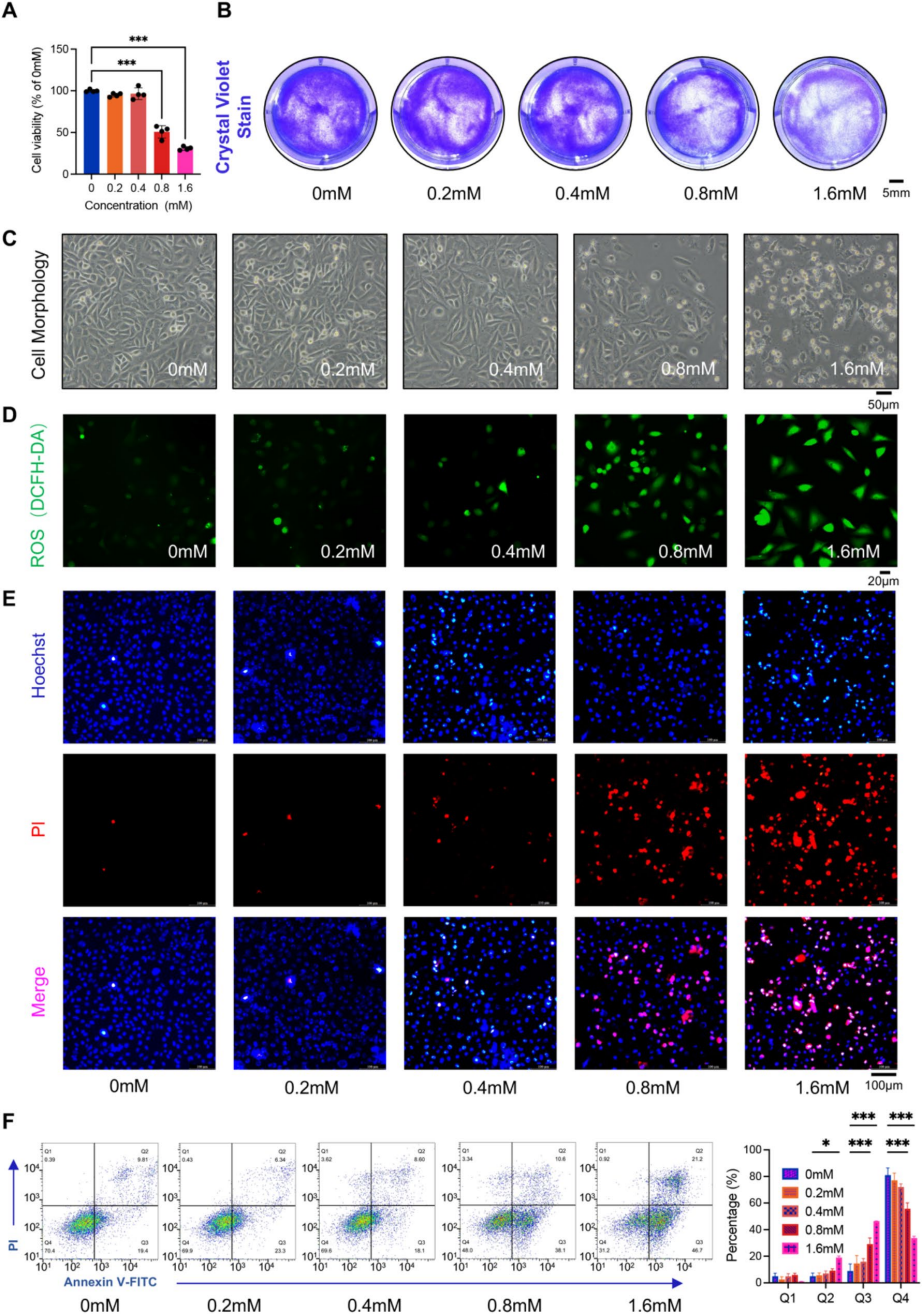
MGO induces VECs death in a dose dependent manner. **A**. HUVECs were treated with different concentrations of MGO for 24 h and the cell viability was detected by CCK-8 (Statistical analysis was performed using one-way ANOVA, followed by Dunnett test, *n* = 4). **B**. Crystal violet staining was performed to evaluate the viability of cells after 24 h of MGO treatment. Scale bar = 5 mm. **C**. After treating cells with different concentrations of MGO for 24 h, the cell morphology was evaluated. Scale bar = 50 μm. **D**. ROS content of HUVECs treated with different concentrations of MGO for 6 h was detected by DCFH-DA staining. Scale bar = 20 μm. **E**. Pyridine iodide (PI) and Hoechst staining analysis of the proportion of living cells (blue) and dead cells (red) in HUVECs after treatment with different concentrations of MGO for 24 h. Scale bar = 100 μm. **F**. Flow cytometry detection of Annexin V and PI positive cells in HUVECs after treatment with different concentrations of MGO (same as A) for 24 h. Statistical analysis showed the percentage distribution of cells within different treatment groups, where Q1 represents Annexin V-negative/PI-positive, Q2 represents Annexin V-positive/PI-positive, Q3 represents Annexin V-positive/PI-negative, and Q4 represents Annexin V-negative/PI-negative (Statistical analysis was performed using two-way ANOVA, followed by Dunnett test, *n* = 3). Data were presented as mean ± SD, *P < 0.05, ***P < 0.001

Treatment of HUVECs with MGO resulted in a marked decrease in cell viability at elevated concentrations of ≥ 0.8 mM (Fig. 1A). Crystal violet staining further demonstrated a gradual reduction in surviving cells as the MGO concentration increased (Fig. 1B).

Morphological analysis revealed that, as MGO concentration increased, the HUVECs transitioned from a tightly arranged cobblestone-like to a rounded shape (Fig. 1C). Additionally, significant intracellular ROS accumulation was observed in the MGO-treated HUVECs at concentrations of 0.8 mM or higher (Fig. 1D), which is suggesting that ROS play a critical role in inducing HUVEC death. Consistent with this, HUVECs that were dead after MGO treatment exhibited substantial PI staining, indicating an intense mode of cell death (Fig. 1E). Flow cytometry further demonstrated that MGO exposure increased the proportion of Annexin V-positive/PI-positive cells, while decreasing the proportion of viable cells (Annexin V/PI-negative) (Fig. 1F). These findings collectively suggest that MGO-induced VEC death is associated with enhanced ROS production and membrane damage.

### MGO triggers pyroptosis in HUVECs via ROS/Caspase-3/GSDME signaling pathway

**Fig. 2:**
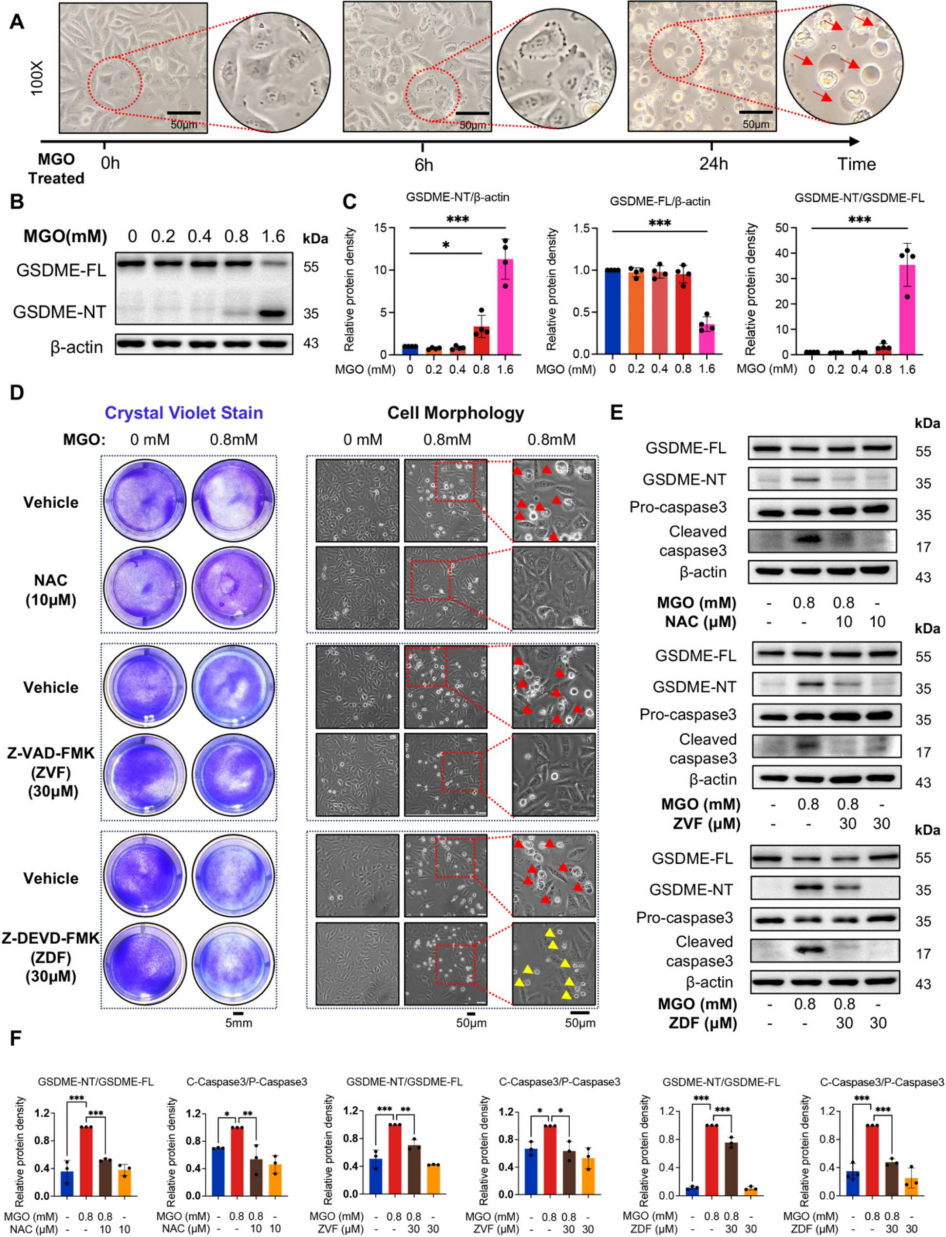
MGO induces HUVECs pyroptosis via ROS/Caspase3/GSDME pathway. **A**. The representative morphology and the phenotype of bubbles of pyroptosis cells (red arrows) after 0.8 mM MGO treatment for HUVECs at 0, 6 and 24 h. Scale bar = 50 μm. **B** and **C**. HUVECs were treated with different concentrations of MGO (0, 0.2, 0.4, 0.8 and1.6 mM) for 24 h, the expression of GSDME-FL and GSDME-NT was detected by Western blotting (**B**). Statistical analysis was performed using Image J (**C**) (Statistical analysis was performed using one-way ANOVA, followed by Dunnett test, *n* = 4). **D**. Crystal violet staining and morphology showed the effect of NAC, Z-VAD-FMK (ZVF), and Z-DEVD-FMK (ZDF) on MGO-induced HUVECs death (red arrow: pyroptosis; yellow arrow: apoptosis). Crystal violet staining: scale bar = 5 mm, and the morphology analysis: scale bar = 50 μm. **E** and **F**. Western blotting was performed to analyze the expression levels of GSDME-FL, GSDME-NT, Pro Caspase3, and Cleaved Caspase3 in different inhibitor groups (E). Statistical analysis was performed using Image J (**F**). (Statistical analysis was performed using one-way ANOVA, followed by Dunnett test, *n* = 3). Data were presented as mean ± SD, *P < 0.05, **P < 0.01, ***P < 0.001 vs. MGO group

To evaluate the impact of MGO treatment on HUVECs, cell morphology was examined under a phase-contrast microscope at various time points following the administration of 0.8 mM MGO. Six hours after treatment, HUVECs exhibited cellular shrinkage, and cell death occurred within 24 h. Additionally, distinct bubble-like protrusions, characteristic of pyroptotic cells, were evident (Fig. 2A), suggesting that MGO treatment induces pyroptosis [19]. Western blotting revealed a significant upregulation of GSDME-NT expression in HUVECs following MGO treatment (Fig. 2B, C). These findings confirm that MGO induces pyroptosis in HUVECs. Given that 1.6 mM MGO caused excessive cell death, while 0.8 mM MGO significantly increased GSDME-NT levels, we selected 0.8 mM MGO for all the subsequent experiments.

To elucidate the specific pathways involved in MGO-induced pyroptosis, we employed several inhibitors to pharmacologically block distinct stages of the pyroptotic process. As previously reported, MGO treatment led to a significant increase in intracellular ROS levels (Fig. 1D). Excessive ROS disrupts the redox balance, thereby activating Caspase-mediated cell death, including Caspase3 activation [35]. Therefore, HUVECs were treated with the ROS inhibitor NAC, the pan-Caspase inhibitor Z-VAD-FMK, and the specific Caspase3 inhibitor Z-DEVD-FMK.

Both NAC and Z-VAD-FMK effectively inhibited MGO-induced cell death and suppressed the characteristic “bubbling” phenomenon associated with pyroptosis (Fig. 2D). Western blotting confirmed that MGO treatment upregulation of GSDME-NT and cleaved Caspase3, whereas pretreatment with NAC and Z-VAD-FMK completely blocked these changes (Fig. 2E, F).

Furthermore, pretreatment of cells with the Caspase3-specific inhibitor Z-DEVD-FMK (ZDF) effectively suppressed the MGO-induced upregulation of cleaved Caspase3 and GSDME-NT (Fig. 2E, F), although it did not fully prevent cell death. Morphologically, pretreatment with Z-DEVD-FMK reduced MGO-induced cellular bubble-like protrusions, replacing them with cell shrinkage, a feature more characteristic of apoptosis (Fig. 2D). Further experiments revealed that, despite Caspase3 inhibition, MGO-induced cell death continued via cleaved Caspase7 mediation (Figure S1A). Collectively, these results suggest that MGO induces pyroptosis in HUVECs through an ROS/Caspase3/GSDME pathway.

### DT-13 prevents MGO-induced HUVEC pyroptosis by inhibiting GSDME-NT production

**Fig. 3:**
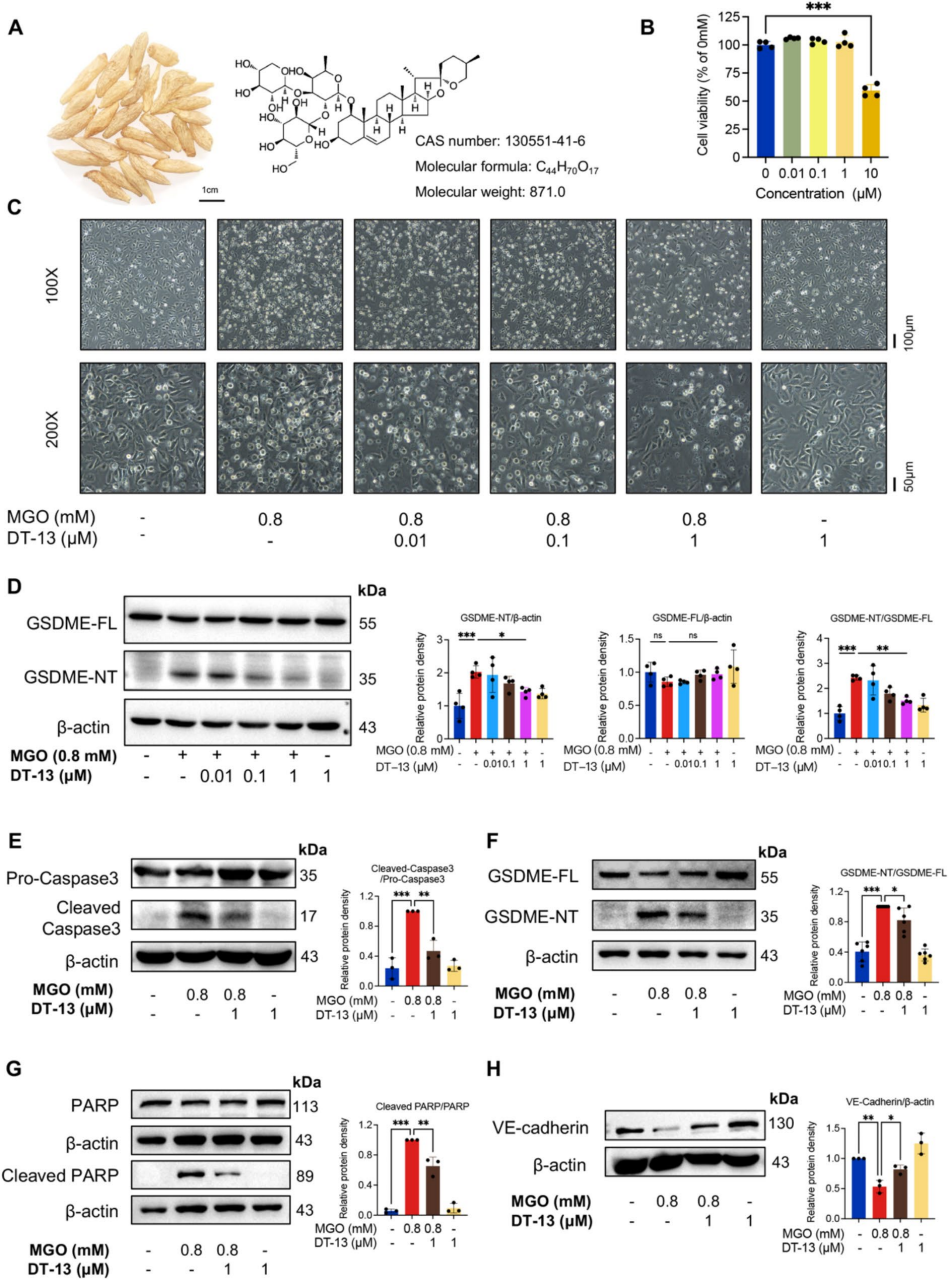
DT-13 inhibited MGO-induced HUVECs pyroptosis by inhibiting GSDME-NT production. **A**. Representative pictures of Ophiopogon japonicus and the structural formula, CAS number, molecular formula, and molecular weight of DT-13. Scale bar = 1 cm. **B**. The cell viability of HUVECs exposed to different concentrations of DT-13 for 24 h was detected by CCK-8 assay and expressed as a percentage of the control group. (Statistical analysis was performed using one-way ANOVA, followed by Dunnett test, *n* = 4). **C**. Representative morphology of HUVECs after pretreatment with 0.01, 0.1, and 1 µM DT-13 for 6 h and exposure to MGO (0.8 mM) for 24 h (white dots were dead cells). Scale bar = 100 μm (top)/50 µm (bottom). **D**. HUVECs were pretreated with DT-13 at varying concentrations for 6 h and exposed to MGO (0.8 mM) for 24 h. Western blotting detected the protein levels of GSDME-FL and GSDME-NT in HUVECs after various treatment groups, and the statistical analysis was performed using Image J. (Statistical analysis was performed using one-way ANOVA, followed by Tukey test, *n* = 4). **E-H**. HUVECs were pretreated with DT-13 (1 µM) for 6 h and exposed to MGO (0.8 mM) for 24 h. Western blotting was employed to detect the protein expressions of Pro-Caspase3, Cleaved Caspase3 (**E**) (statistical analysis was performed using one-way ANOVA, followed by Tukey test, *n* = 3), GSDME-FL, GSDME-NT (**F**) (Statistical analysis was performed using one-way ANOVA, followed by Tukey test, *n* = 6), PARP and Cleaved PARP **(G)** (Statistical analysis was performed using one-way ANOVA, followed by Tukey test, *n* = 3), and VE-Cadherin (**H**) (Statistical analysis was performed using one-way ANOVA, followed by Tukey test, *n* = 3). The statistical analysis was performed using Image J. Data were presented as mean ± SD, *P < 0.05, **P < 0.01, ***P < 0.001

The natural product DT-13, classified as a member of the saponins (Fig. 3A), exhibits diverse pharmacological effects, including anti-inflammatory, antioxidant, and insulin-sensitizing properties [36, 37]. The safe concentration of DT-13 was first determined by a CCK-8 assay, showing that DT-13 at concentrations below 1 µM is non-toxic to VECs (Fig. 3B). Notably, pretreatment with DT-13 effectively mitigated MGO-induced HUVECs death and the upregulation of GSDME-NT in a dose-dependent manner (Fig. 3C, D). Furthermore, we found that pretreatment with DT-13 reduced the expression of cleaved Caspase3, which subsequently led to a decrease in the generation of GSDME-NT (Fig. 3E, F). Thus, the functional activity of DT-13 is like that of the Caspase 3 inhibitor Z-DEVD-FMK but differs in that DT-13 could inhibit the expression of cleaved Caspase7 induced by MGO (Figure S1B).

To clarify DT-13 specifically targets Caspase3 instead of GSDME, we examined the cleavage levels of Caspase3 downstream proteins, such as GSDME and PARP. The cleavage of both GSDME and PARP was significantly inhibited by DT-13 (Fig. 3F, G). This finding supports that DT-13 effectively suppresses MGO-induced pyroptosis by inhibiting cleaved Caspase3 and GSDME-NT. Additionally, DT-13 treatment was found to rescue the decreased expression of VE-Cadherin caused by MGO treatment (Fig. 3H). However, despite the protective effect of DT-13 on VECs, it failed to completely abrogate MGO-induced cell death. This could be attributed to the involvement of other modes of death.

### DT-13 induces PAT by promoting apoptotic bodies formation

**Fig. 4:**
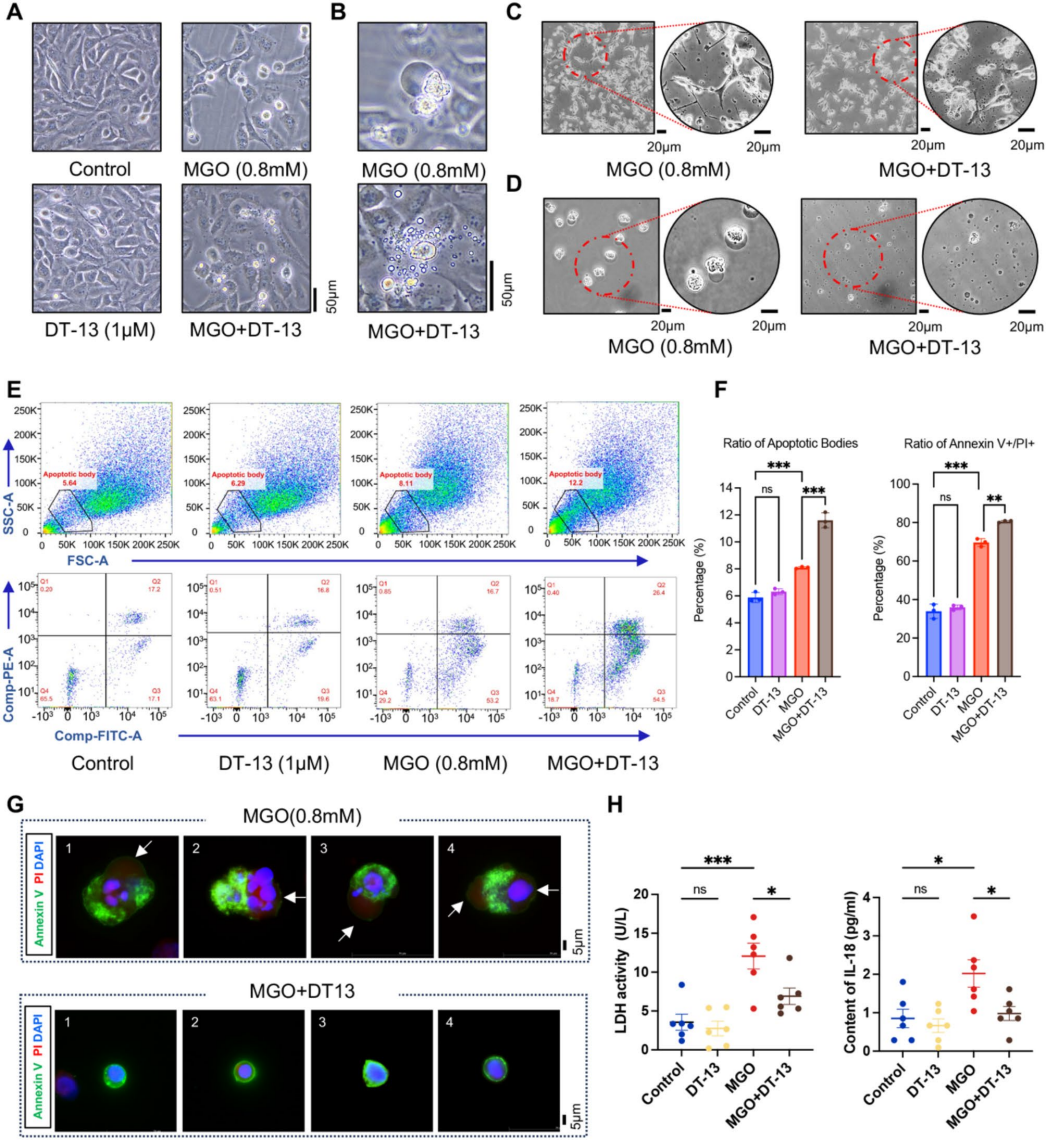
DT-13 converts MGO-induced pyroptosis to apoptosis by promoting the formation of apoptotic bodies. **A**. The representative morphology of HUVECs in each group after pretreatment with DT-13 (1 μM) for 6 h and then exposure to MGO (0.8 mM) for 24 h. Scale bar = 50 μm. **B**. The morphology of dead cells in MGO-induced pyroptosis (top) and in apoptosis following DT-13 pretreatment (bottom). Scale bar = 50 μm. **C** and **D**. The number of apoptotic bodies in the intercellular space (C) and cell culture medium supernatant (D) was evaluated under MGO induction with and without DT-13. Scale bar = 20 μm. **E**. Flow cytometry was used to detect the proportion of apoptotic bodies in each group (top), and the proportion of Annexin V and PI staining in apoptotic bodies in each group (bottom). **F**. Statistical analysis results of apoptotic body content and statistical analysis results of Annexin V-positive/PI-positive in apoptotic bodies (Statistical analysis was performed using one-way ANOVA, followed by Tukey test, *n* = 3). **G**. Fluorescence staining of Annexin V (green), PI (red), and DAPI (blue) in pyroptosis cells and apoptotic bodies. The white arrows indicate swollen cell membranes in pyroptotic cells. **H.** The LDH and IL-18 levels in the supernatants of cell cultures from each group were quantified by LDH Kit and IL-18 ELISA Kit (Statistical analysis was performed using one-way ANOVA, followed by Tukey test, *n* = 6). Data were presented as mean ± SD, *P < 0.05, **P < 0.01, ***P < 0.001

Under phase-contrast microscopy, pretreatment with DT-13 induced significant morphological changes in cells exposed to MGO-induced cell dead. Specifically, the number of pyroptotic cells with bubble-like protrusions decreased, while the number of apoptotic cells increased (Fig. 4A, B). In these apoptotic cells, extracellular vesicles of varying sizes aggregated around the shrunken nucleus, forming apoptotic bodies that dispersed into the medium under external forces, such as the physical agitation of the culture dish (Fig. 4C). This phenomenon was more evident in the culture medium. As shown in Fig. 4D, unlike the culture medium from the MGO group, which contained many pyroptotic cells, the medium from the DT-13-pretreated group showed a significantly higher number of apoptotic bodies (diameter approximately 1–5 μm).

Flow cytometry analysis further demonstrated that DT-13 pretreatment significantly increased the proportion of apoptotic bodies among the MGO-induced dead cells, as well as the ratio of Annexin V-positive/PI-positive cells in these apoptotic bodies (Fig. 4E, F). Immunofluorescence staining revealed that MGO-induced cell death resulted in cell swelling, with Annexin V-positive membranes compressed to one side. The opposite side thinned and developed bubble-like structures that were prone to rupture, facilitating the release of DNA, nucleic acids, and other cellular contents. In contrast, DT-13 pretreatment promoted the formation of apoptotic bodies in the damaged cells, with Annexin V-positive membranes fully encapsulating cellular contents, thereby preventing content leakage (Fig. 4G).

Additionally, the levels of LDH and IL-18 in the supernatant significantly increased following MGO treatment, whereas DT-13 pretreatment effectively reduced the release of these inflammatory substances induced by MGO (Fig. 4H). These findings suggest that DT-13 can convert MGO-induced pyroptosis into apoptosis, thereby mitigating the inflammatory response triggered by the release of cellular contents from the pyroptotic cells.

### DT-13 promotes cytoskeleton rearrangement-related apoptotic body formation through MLC2 phosphorylation

**Fig. 5:**
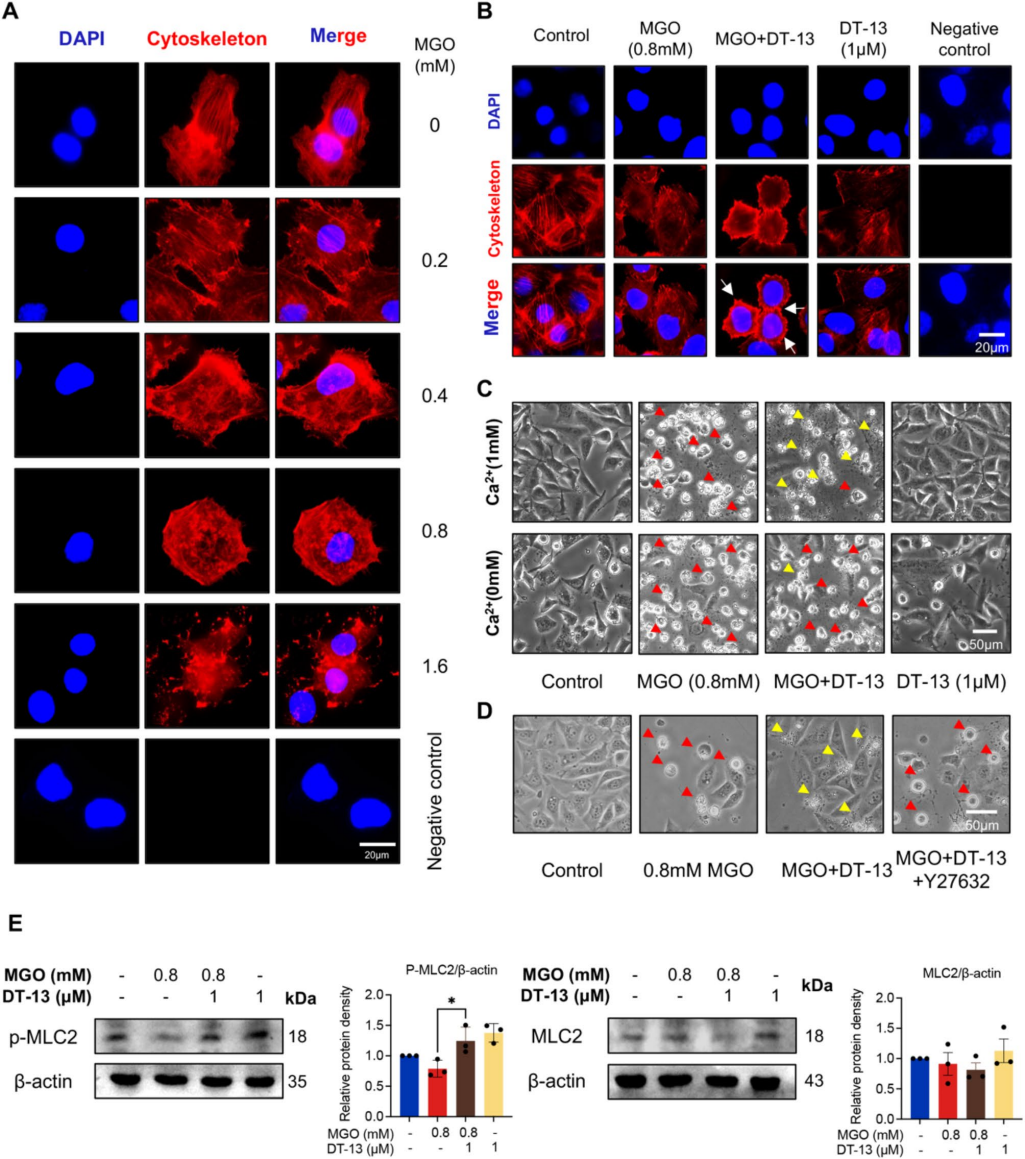
DT-13 promoted cytoskeletal rearrangement and apoptotic body formation via phosphorylation of MLC2. **A**. Immunofluorescence showed the cytoskeletal morphological changes of HUVECs exposed to different concentrations of MGO (0, 0.2, 0.4, 0.8 and 1.6 mM) for 24 h. Rhodamine-Phalloidin (red) stains the cytoskeleton, DAPI (blue) stains the nucleus. In the negative control group, rhodamine-phalloidin was not used. Scale bar = 20 μm. **B**. Representative immunofluorescence staining of cytoskeleton in HUVECs after pretreatment with DT-13 (1 µM) for 6 h and exposure to MGO (0.8 mM) for 24 h. Rhodamine-Phalloidin (red) stains the cytoskeleton, DAPI (blue) stains the nucleus (peripheral ring: white arrow). In the negative control group, rhodamine-phalloidin was not used. Scale bar = 20 μm. **C**. The representative morphological images of HUVECs in different treatment groups (same as B) with or without Ca^2+^. (Red arrows: pyroptosis cells, yellow arrows: apoptosis cells). Scale bar = 50 μm. **D**. Effect of the ROCK inhibitor Y27632 (5 µM, 2 h) on the formation of apoptotic bodies induced by DT-13. (Red arrows: pyroptosis cells, yellow arrows: apoptosis cells). Scale bar = 50 μm. **E** Western blotting was performed to detect the protein levels of p-MLC2 and MLC2 in different treatment groups, and statistical analysis was performed using Image J (Statistical analysis was performed using one-way ANOVA, followed by Tukey test, *n* = 3). Data were presented as mean ± SD, *P < 0.05

To investigate how DT-13 induces the formation of apoptotic bodies, we first examined its impact on cytoskeletal arrangement given the crucial role of the cytoskeleton in this process. With increasing concentrations of MGO, the originally well-organized cytoskeleton became disorganized and degraded (Fig. 5A). However, this damage was ameliorated after DT-13 treatment, as evidenced by their improved cytoskeletal reorganization and the formation of peripheral rings (Fig. 5B). It is well-documented that the disappearance of cellular stress fibers and the reorganization of actin into peripheral rings are key events in apoptotic body formation [38]. Thus, our observations here strongly suggest that cytoskeletal rearrangement plays a significant role in DT-13-induced apoptosis.

Additionally, we observed that the formation of apoptotic bodies induced by DT-13 was significantly inhibited in the absence of Ca^2+^ (Fig. 5C). Given that Ca^2+^ cooperates with ROCK via Calmodulin (CaM) to enhance the phosphorylation of the cytoskeletal protein MLC2, HUVECs were treated with the ROCK inhibitor Y27632. This treatment significantly inhibited the apoptotic effect induced by DT-13 (Fig. 5D). Moreover, compared to the MGO-only group, DT-13 pretreatment significantly enhanced the phosphorylation level of MLC2 (Fig. 5E). These results strongly suggest that DT-13 promotes apoptotic body formation in MGO-treated HUVECs through the Ca^2+^/ROCK/p-MLC2 pathway.

### DT-13 inhibits MGO-induced cleavage of non-muscle myosin heavy chain (NMMHC IIA)

**Fig. 6:**
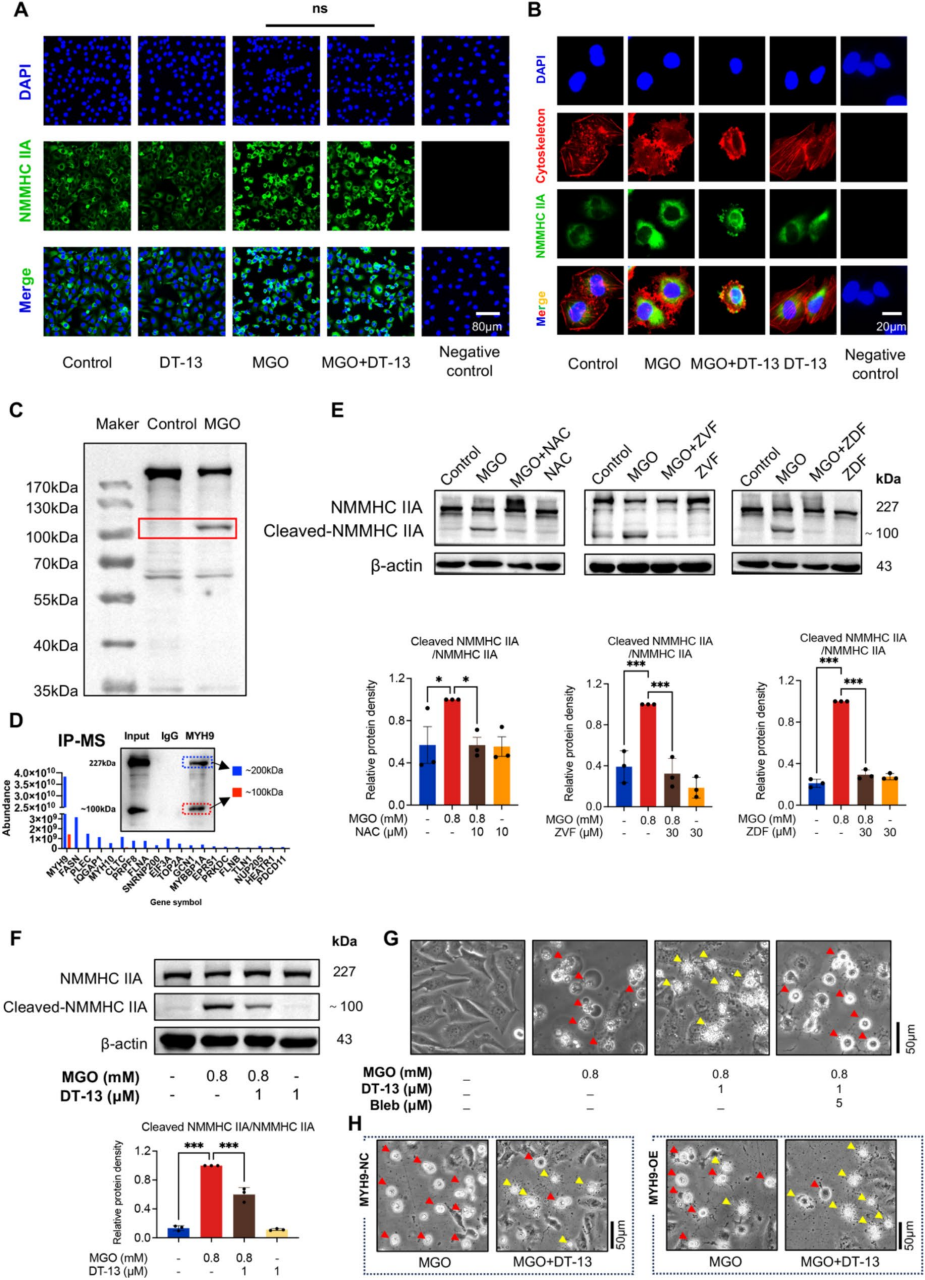
DT-13 inhibited MGO-induced NMMHC IIA cleavage and increased apoptotic body formation via NMMHC IIA. **A**: The expression of NMMHC IIA (green) in each group was detected and analyzed by immunofluorescence. DAPI (blue) stains the nucleus. In the negative control group, the primary antibody against MYH9 was not used. The relative expression level was analyzed using fluorescence intensity (Statistical analysis was performed using t-test, followed by D’Agostino-Pearson omnibus test and Brown-Forsythe test, *n* = 6, “ns” indicates no significant statistical difference). Scale bar = 80 μm. **B**. The cytoskeleton morphology and the location of NMMHC IIA in each group were analyzed by immunofluorescence. DAPI (blue) stains the nucleus, rhodamine-Phalloidin (red) stains the cytoskeleton, NMMHC IIA is visualized in green. Yellow indicating the colocalization of NMMHC IIA with the cytoskeleton. **C**. Western blotting was performed to detect the cleavage of NMMHC IIA protein in HUVECs treated with MGO (the red box is Cleaved-NMMHC IIA). **D** The IP-MS experiment showed that the 100 kDa sized band is Cleaved-NMMHC IIA. **E**. Western blotting was performed to detect the effects of NAC, Z-VAD-FMK (ZVF), and Z-DEVD-FMK (ZDF) on the upregulation of Cleaved-NMMHC IIA induced by MGO. Statistical analysis was performed using Image J (Statistical analysis was performed using one-way ANOVA, followed by Dunnett test, *n* = 3). **F**. Western blotting was conducted to analyze the alleviation of MGO-induced upregulation of Cleaved-NMMHC IIA by DT-13 and the statistical analysis was performed using Image J (Statistical analysis was performed using one-way ANOVA, followed by Tukey test, *n* = 3). **G**. Effects of Bleb (5 µM, 2 h) on the ability of DT-13 to induce apoptotic body formation (red arrows: pyroptosis cells, yellow arrows: apoptotic cells) Scale bar = 50 μm. **H**. Effect of MYH9 overexpression on DT-13-induced apoptotic body formation. The left is negative control adenovirus group, and the right is MYH9 overexpression adenovirus group (red arrows: pyroptosis cells, yellow arrows: apoptotic cells), Scale bar = 50 μm. Data were presented as mean ± SD, *P < 0.05, **P < 0.01, ***P < 0.001

NMMHC IIA is known to facilitate extracellular vesicle formation by interacting with cytoskeletal proteins such as microfilaments, it utilizes ATP hydrolysis-driven myosin movement to generate force, contributing to the formation of vesicles, including apoptotic bodies [39–42]. In this study, NMMHC IIA protein expression significantly increased in both the MGO-only and DT-13 pre-treated groups, with no significant difference between them (Fig. 6A). However, further investigation revealed differences in the distribution of NMMHC IIA between the two groups of cells. Specifically, MGO treatment caused NMMHC IIA protein to disperse throughout the cytoplasm, rather than clustering around the cytomembrane. In contrast, in DT-13 pre-treated cells, NMMHC IIA localized preferentially at the cytomembrane and around apoptotic bodies, co-localizing with the cytoskeleton and accompanied by evident cytoskeletal rearrangement (Fig. 6B). These findings suggest that NMMHC IIA is involved in DT-13-induced apoptotic body formation.

Additionally, an enhanced cleavage of NMMHC IIA was observed following MGO treatment. Specifically, the Western blotting revealed a cleavage fragment with a molecular weight (MW) of approximately 100 kDa, significantly lower than the original 224 kDa MW of NMMHC IIA. Protein mass spectrometry further confirmed this fragment as a cleavage product of NMMHC IIA (Fig. 6D; Figure S2). Based on the MW and peptide data (Table S1, S2), we speculate that the cleavage occurs at the head-tail junction site of NMMHC IIA, leading to a loss of structural integrity and affecting its functionality. These data also help interpret the morphological changes observed in MGO-treated HUVECs.

To elucidate the molecular pathway of NMMHC IIA cleavage induced by MGO, we employed inhibitors NAC, Z-VAD-FMK, and Z-DEVD-FMK to examine the involvement of the ROS/Caspase pathway. Our results showed that pretreatment with NAC, Z-VAD-FMK, and Z-DEVD-FMK not only significantly inhibited MGO-induced pyroptosis in HUVECs but also suppressed the production of cleaved-NMMHC IIA (Fig. 6E). This suggests that MGO-induced cleavage of NMMHC IIA induced by MGO is mediated through the ROS/Caspase pathway, with Caspase3 playing a significant role in this process. Furthermore, DT-13 significantly inhibited MGO-induced cleavage of NMMHC IIA (Fig. 6F), suggesting that NMMHC IIA contributes to DT-13-induced apoptotic body formation.

To validate this hypothesis, we used Bleb a potent inhibitor of ATPase activity in NMMHC IIA, and an NMMHC IIA overexpression adenovirus (MYH9-OE) to assess the role of NMMHC IIA. Our results showed that Bleb inhibited DT-13-induced apoptotic body formation in HUVECs (Fig. 6G). However, overexpression of NMMHC IIA did not inhibit MGO-induced pyroptosis in HUVECs but significantly enhanced DT-13-triggered apoptotic body formation (Fig. 6H). Collectively, these data demonstrate that although MGO upregulated NMMHC IIA expression, its activity was compromised by Caspase3-mediated cleavage, thereby inhibiting apoptotic body formation. DT-13 effectively prevents MGO-induced cleavage of NMMHC IIA, promoting its role in apoptotic body formation and transforming the cell death mode from pyroptosis to apoptosis.

### DT-13 alleviates MGO-induced vascular lesions and dysfunction by protecting VECs

To further investigate the protective effect of DT-13 on MGO-induced vascular damage at the tissue and organ levels, we employed mouse ex vivo mouse vascular rings, human pluripotent stem cell-derived hVOs, and a mouse animal model to assess its protective impact.

**Fig. 7:**
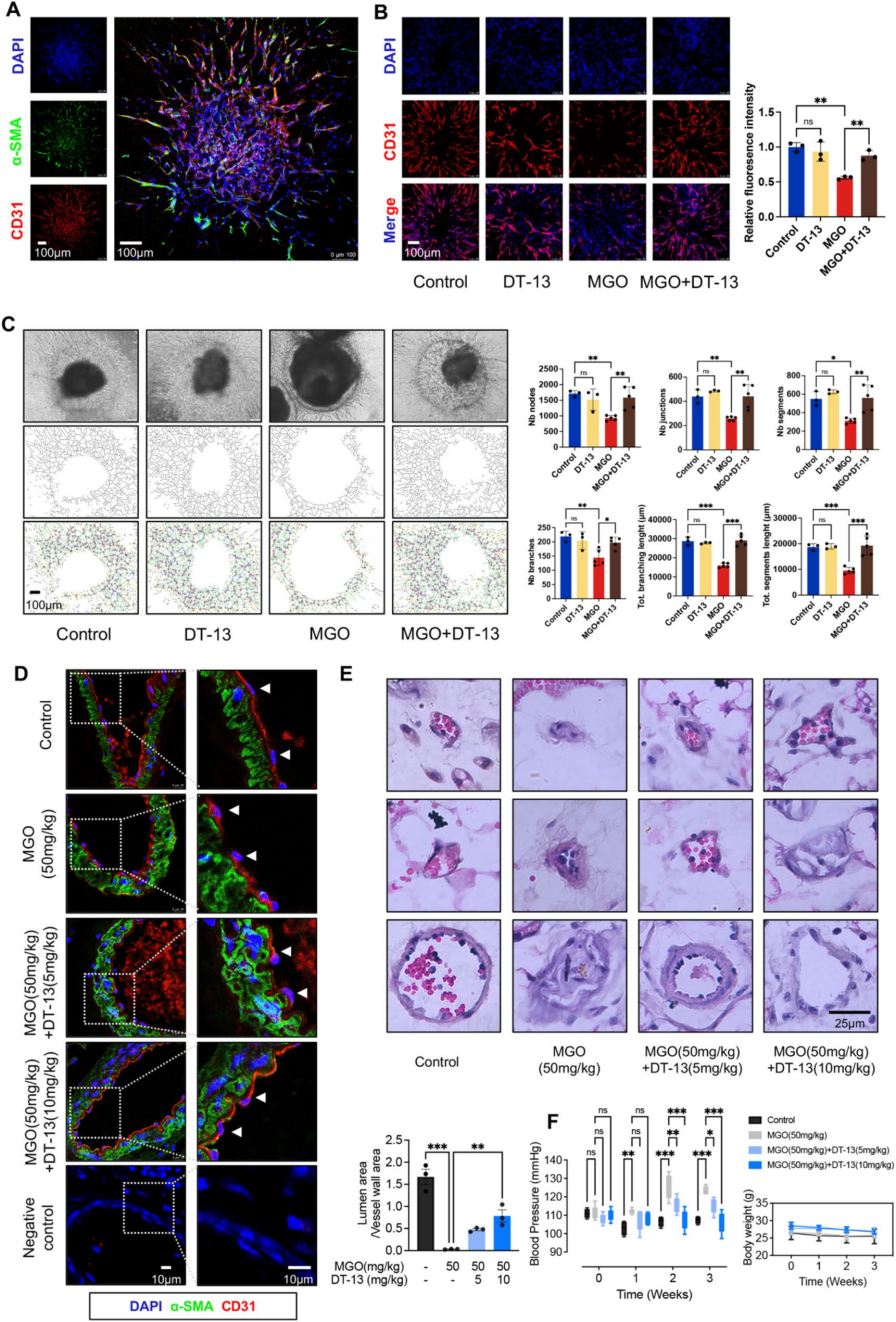
DT-13 alleviated loss of VECs, vascular remodeling, and hypertension induced by MGO. **A**. The immunofluorescence staining results revealed a complete hVOs structure: the nucleus is blue, VSMCs are green, and VECs are red. Scale bar = 100 μm. **B**. Immunofluorescence staining was performed to observe the number of VECs in the different treatment groups of hVOs. DAPI (blue) stains the nucleus and CD31 (red, Alexa Fluor^®^ 647-conjugated)) represents the endothelial cells. Quantitative analysis of the intensity of red fluorescence was performed using Image J (Statistical analysis was performed using one-way ANOVA, followed by Tukey test, *n* = 3). Scale bar = 100 μm. **C**. After pretreating hVOs with 1 µM DT-13 for a duration of 6 h, the organoids were exposed to MGO (0.8 mM) for a period of 24 h. Collect hVOs from different treatment groups. Subsequently, capture photographs of the organoids (top) and use Image J software to binarize the vascular network (middle). Analyze the binarized vascular network using the AngioTool plug-in (bottom). Statistically analyze the number of nodes (Nb nodes), junctions (Nb junctions), segments (Nb segments), branches (Nb branches), total length of segment (Tot. segments length) and branches (Tot. branches length) (Statistical analysis was performed using one-way ANOVA, followed by Tukey test, *n* = 3–5). Scale bar = 100 μm. **D**. Characterization of α-SMA (green) and CD31 (red) in mouse mesenteric vessels by immunofluorescence. DAPI (blue) stains the nucleus. In the negative control group, neither the primary antibody against α-SMA nor the primary antibody against CD31 was used, scale bar = 10 μm. **E**. H&E staining of mouse mesenteric vessels showing the morphology of mouse mesenteric vessels in each group. Three kinds of vessels with different diameters were selected in each group, and the ratio of lumen area to vessel wall area was statistically analyzed (*n* = 3). Scale bar = 25 μm. **F**. Statistical analysis was conducted on the systolic blood pressure (SBP) of each group of mice, as well as on the changes in body weight among different groups (Statistical analysis was performed using two-way ANOVA, followed by Dunnett test, *n* = 3–5). Data were presented as mean ± SD, *P < 0.05, **P < 0.01, ***P < 0.001

First, ex vivo mouse vascular rings were incubated with MGO for 20 min, and their response to acetylcholine stimulation was significantly lower compared to the control group (no MGO group). In contrast, no significant change in response was observed when the rings were stimulated with sodium nitroprusside, indicating that MGO primarily damages VECs rather than vascular smooth muscle cells (VSMCs) (Figure S3). This result was corroborated using the hVOs model system. Following the induction protocol from J. M. Penninger’s laboratory [34], we successfully prepared hVOs containing both VECs and VSMCs (Fig. 7A). Immunofluorescence staining revealed that MGO treatment reduced the number of CD31-positive cells in the hVOs and inhibited angiogenesis. However, DT-13 pretreatment significantly alleviated this inhibitory effect (Fig. 7B). Specifically, after 24 h of MGO treatment, the number of nodes, connections, segments, and branches in the hVOs decreased significantly, and the total length of segments and branches in the vascular network was also reduced, as analyzed using Image J software (Fig. 7C). In contrast, DT-13 pretreatment effectively restored these parameters, demonstrating its protective effect.

Next, histological examination in a mouse model revealed that in the MGO treatment caused swelling of mesenteric VECs, loose cellular connections, and cell shedding (Fig. 7D), along with characteristics of diabetes-like vascular lesions, including severe vascular stenosis and lumen occlusion (Fig. 7E). However, after DT-13 treatment significantly alleviated these pathological changes. Additionally, DT-13 attenuated the production of GSDME-NT induced by MGO in mouse mesenteric vessels, despite using antibodies with human species reactivity (Figure S4).

Finally, to further determine whether the vascular protective effect of DT-13 could improve vascular function, blood pressure was regularly monitored in all groups of mice. The results showed that the blood pressure in the model group increased steadily over time, while a significant decrease in blood pressure was observed in the DT-13 treated mice (Fig. 7F). Since no significant differences were observed in body weight (Fig. 7F), heart weight (Figure S5A), or cardiac function (Figure S5B, C) between the two groups, the observed differences in blood pressure changes can be attributed to the vascular protective effect of DT-13. These findings support the conclusion that DT-13 alleviates MGO-induced vascular dysfunction.

## Discussion

MGO, a byproduct of glucose metabolism, is a known direct cause of DVCs [24–26]. However, the role of inflammatory cell death, particularly pyroptosis, in MGO-induced VEC damage remains unclear. This study demonstrates that MGO induces GSDME-dependent pyroptosis in HUVECs, as evidenced by Annexin V/PI positivity, increased inflammatory markers, and GSDME-NT release. Furthermore, DT-13, mitigates MGO-induced pyroptosis, reduces inflammation, and promotes apoptosis. DT-13 effectively converts MGO-induced inflammatory cell death into a non-inflammatory form, thus protecting blood vessels. These findings offer valuable insights for managing high glucose-induced VEC damage by modulating cell death pathways.

Pyroptosis is a form of PCD with specific physiological functions [43]. However, excessive pyroptosis, especially in VECs, can result in vascular injury and dysfunction, contributing to diseases such as pulmonary hypertension [44] and ischemic conditions [45, 46]. Research has shown that pyroptosis plays a critical role in diabetes-related vascular diseases, including diabetic retinopathy [47] and atherosclerosis [48]. Although MGO accumulation has been shown to activate Caspase3 in VECs, leading to cell death [19], it remains unclear whether MGO triggers GSDME-dependent pyroptosis via Caspase3 activation. Our study is the first to clarify this issue, providing new evidence for the role of pyroptosis in diseases associated with Caspase3 activation.

Interestingly, MGO-induced pyroptosis can be converted into apoptosis by DT-13, as evidenced by the suppression of pyroptosis markers (such as GSDME-NT, IL-18, and LDH release) and an increase in the proportion of apoptotic bodies. The increase in apoptotic body formation can facilitate the transition of the cell death process toward apoptosis, a process in which NMMHC IIA plays a critical role [39–42, 49]. Our study found that DT-13 induces apoptotic body formation via NMMHC IIA, while MGO also upregulates NMMHC IIA in HUVECs, a phenomenon observed in various disease models [50, 51]. The exact reasons for this upregulation and its significance in disease states are not fully understood. NMMHC IIA may play a role in signal transduction [51]. Nevertheless, our study demonstrates that MGO-induced upregulation of NMMHC IIA is cleaved by Caspase3, leading to a loss of its function, which prevents apoptotic body formation and results in membrane rupture. This was further confirmed by our experiments with the Caspase3 inhibitor Z-DEVD-FMK. Pretreatment with Z-DEVD-FMK significantly inhibited MGO-induced NMMHC IIA cleavage, resulting in cell shrinkage and apoptotic bodies formation. These results strongly support the critical role of the intact NMMHC IIA molecule in promoting the formation of apoptotic bodies.

Cell death activation is crucial for cellular homeostasis, but different modes of cell death can have varied effects on cell fate [1]. MGO-induced cell death is primarily triggered by mitochondrial oxidative stress, leading to the activation of caspase activity, which eliminates damaged cells and maintains tissue and organ function [31, 52]. This mechanism is not only present in diabetes-related diseases but also in hypoxia, reperfusion, and even COVID-19-induced vascular diseases [45–47, 53], highlighting the common characteristics of these conditions and the clinical significance of this study. Therefore, investigating the associations and transitions between different modes of cell death, especially pyroptosis and apoptosis, is essential for the treatment of clinical diseases. For instance, a report by Wang et al. demonstrated that overexpression of GSDME converts apoptosis induced by chemotherapeutic drugs such as doxorubicin (DOX) and 5-fluorouracil (5-FU) into pyroptosis, resulting in intense inflammatory cell death and enhanced therapeutic outcomes in cancer treatment [19]. Furthermore, recent studies have shown that palmitoylation inhibitors can suppress the occurrence of pyroptosis, converting ROS-induced pyroptosis into apoptosis and reducing inflammatory damage [54, 55]. These findings support the hypothesis that cell death modalities are modifiable. In our study, we found that DT-13 effectively converts MGO-induced pyroptosis in VECs into apoptosis, further confirming the modifiable nature of cell death pathways.

DT-13 has demonstrated significant vascular protective effects and low toxicity in previous studies. Research indicates that DT-13 can mitigate TNF-α-induced vascular inflammation [56], and administration to Sprague-Dawley rats at a dosage of 360 mg/kg for 90 days did not cause hematological, biochemical, or histopathological abnormalities [36]. Our study further supports these findings, showing that DT-13 treatment significantly alleviates MGO-induced diabetic vascular damage in a mouse model. This is evidenced by reduced endothelial cell injury, alleviated vascular lumen narrowing, and inhibition of elevated blood pressure. Moreover, DT-13 did not cause any side effects on the body weight or cardiac function in the mice. We believe that DT-13 promoting PAT in VECs has a significant effect in this process. The reduction of GSDME-NT in the mesenteric artery of mice indicates that DT-13 inhibits pyroptosis in vivo, while in vitro results show that DT-13 alleviates the release of inflammatory factors in MGO-induced VECs, which is crucial for smooth muscle-induced vascular remodeling [53, 57]. However, we have overlooked the fact that VSMCs are also important for vascular remodeling and blood pressure regulation [44, 58–60], which should be further explored in future studies.

It is worth mentioning that traditional Chinese medicine formula “Sha Shen Mai Dong Decoction”, which contains DT-13, has been proven effective in the treatment of pneumonia caused by COVID-19 [37, 53, 61]. Combining the aforementioned studies on the 90-day safety of DT-13 in animal experiments [36] with the results of our research indicates that DT-13 is a promising natural compound for the treatment of inflammatory vascular diseases. Extending its administration period or combining it with appropriate anti-inflammatory drugs may further enhance its therapeutic effects. Overall, these findings suggest that DT-13 has the potential to be developed into a clinical drug. However, clinical translation of DT-13 faces several challenges. More preclinical studies are needed, particularly in pharmacokinetics, safe dosing, and bioavailability, to establish the efficacy and safety of DT-13. Additionally, since natural products often target multiple pathways, it is essential for clinical translation to clarify the interactions between these targets and their potential synergistic effects on efficacy. Therefore, exploring the mechanisms of action and clinical applications of DT-13 will provide a crucial foundation and guidance for the development of natural products in vascular diseases.

## Conclusion

**Fig. 8:**
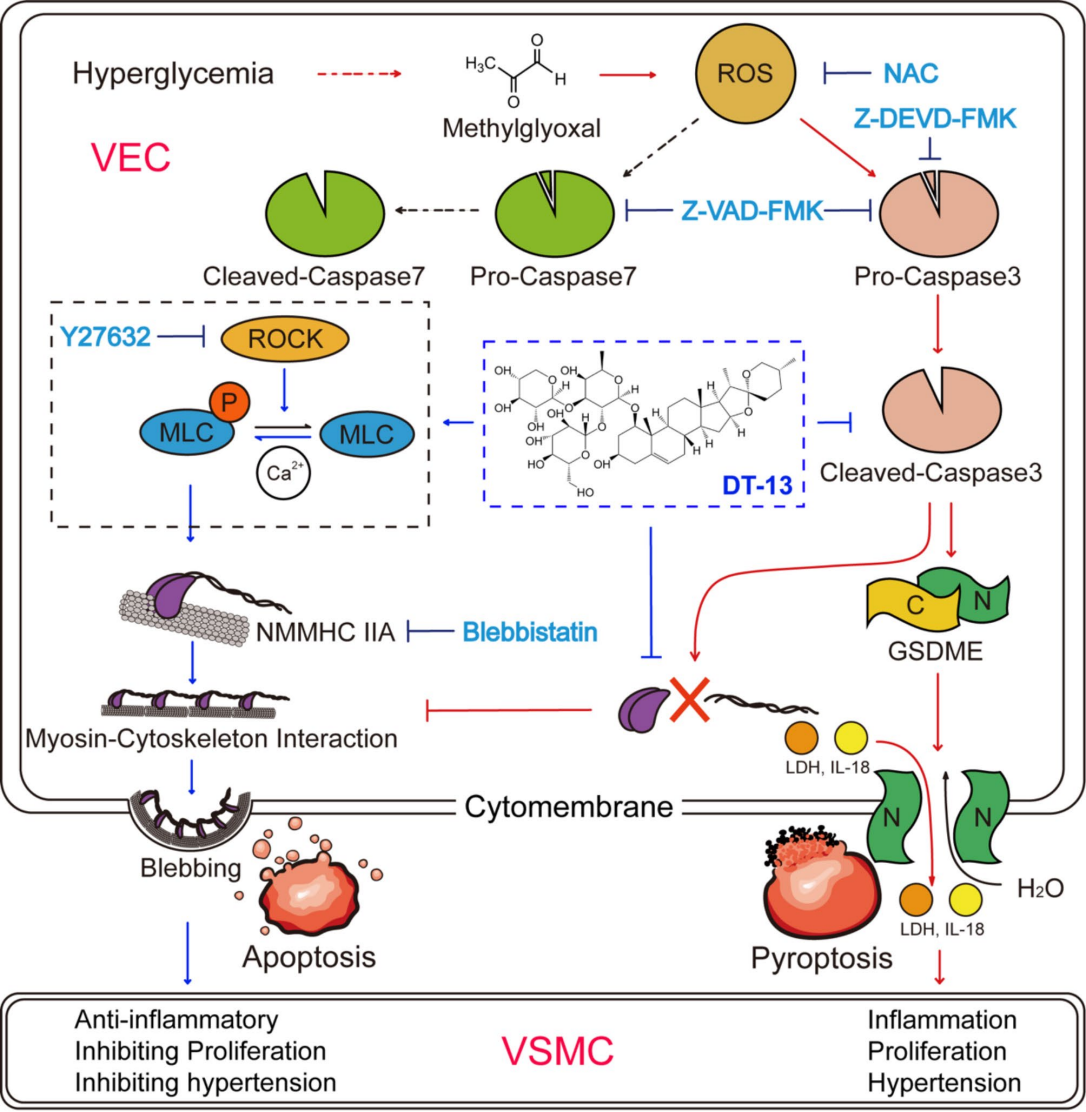
Schematic representation of the mechanism underlying DT-13-mediated inhibition of MGO-induced pyroptosis in HUVECs and promotion of apoptotic body formation. The red line represents the MGO pathway. The black dotted line represents the alternative pathway of MGO upon Caspase3 inhibition. The blue line represents the DT-13 pathway. The Bright blue font represents the action of inhibitors

In conclusion, our study reveals that MGO induces endothelial cell pyroptosis via the ROS/Caspase3/GSDME pathway, leading to cell rupture and the release of inflammatory factors. The natural product DT-13 modulates cytoskeletal rearrangement in endothelial cells through NMMHC IIA by promoting apoptotic bodies formation. It achieves this by inhibiting Caspase3-mediated cleavage of GSDME and NMMHC IIA and inducing MLC2 phosphorylation via ROCK (Fig. 8). This mechanism effectively shifts MGO-induced endothelial cell death from pyroptosis to apoptosis, thereby mitigating MGO-induced vascular damage. These findings are instrumental in exploring therapeutic strategies targeting cell death transformation in cardiovascular diseases and underscore the protective potential of natural products. Moreover, they provide valuable insights for drug development aimed at preventing and treating vascular complications associated in diabetes.

## Electronic supplementary material

Below is the link to the electronic supplementary material.

Supplementary Material 1

Supplementary Material 2

## Data Availability

The data underlying this article will be shared on reasonable request to the corresponding author.

## Abbreviations

VECs: Vascular endothelial cells
MGO: Methylglyoxal
GSDME: Gasdermin E
GSDMD: Gasdermin D
DT-13: Saponin monomer 13 of dwarf lilyturf tuber
NMMHC IIA: Non-muscle myosin heavy chain IIA
MLC2: Myosin light chain 2
p-MLC2: Phospho-myosin light chain 2
PCD: Programmed cell death
MOMP: Mitochondrial outer membrane permeabilization
Apaf-1: Apoptotic protease activating factor-1
DVCs: Diabetic vascular complications
FBS: Fetal bovine serum
hESCs: Human embryonic stem cell line
β-me: β-mercaptoethanol
ROS: Reactive oxygen species
PI: Propidium iodide
NAC: N-Acetylcysteine
DCFH-DA: 2’,7’-dichlorodihydrofluorescein diacetate
eNOS: Endothelial nitric oxide synthase
PBS: Phosphate-buffered saline
SBP: Systolic blood pressure
OD: Optical density
LDH: Lactate dehydrogenase
IL-18: Interleukin 18
MYH9: Myosin heavy chain 9
MOI: Multiplicity of infection
hVOs: Human vascular organoids
OCT: Optimal cutting temperature compound
H&E staining: Hematoxylin and eosin staining
HUVECs: Human umbilical vein endothelial cells
ROCK: Rho associated coiled-coil containing protein kinase
CaM: Calmodulin
MW: Molecular weight
Bleb: Blebbistatin
VSMCs: Vascular smooth muscle cells
DOX: Doxorubicin
5-FU: 5-fluorouracil
PAT: Pyroptosis-apoptosis transition
