# Supplementary material for: Promoting the transition from pyroptosis to apoptosis in endothelial cells: a novel approach to alleviate methylglyoxal-induced vascular damage
Source: J Transl Med. 2025 Feb 10;23:170. doi: 10.1186/s12967-025-06195-x (PMC11809013; doi:10.1186/s12967-025-06195-x)
Supplement: Supplementary file 1 — Supplementary Material 1 [file 12967_2025_6195_MOESM1_ESM.docx]

**Supplementary materials**

**Promoting the Transition from Pyroptosis to Apoptosis in Endothelial Cells: A Novel Approach to Alleviate Methylglyoxal-Induced Vascular Damage**

*Ruqiang Yuan^1,2*^, Hu Xu^3*^, Mingqi Wang^1*^, Lina Guo^1^, Yang Yao^1^, Xiaoru Zhang^1^, Xiuli Wang^1#^*

1. College of Basic Medical Science, Dalian Medical University, Dalian, China, 116044.

2. Advanced Institute for Medical Sciences, Dalian Medical University, Dalian, China, 116044.

3. Health Science Center, East China Normal University, Shanghai, China, 200241.

* These authors contributed equally to this work.

# Corresponding Author: Xiuli Wang, Email: wangxl01@dmu.edu.cn, College of Basic Medical Science, Dalian Medical University, Dalian, China, 116044.

**Supplementary materials and methods**

**Reagents**

Phenylephrine (Phe) (BP284), Acetylcholine (ACh) (A2661), and Sodium nitroprusside (SNP) (71778) were obtained from Sigma. BeaverBeadsTM Protein A/G Immunoprecipitation Kit (22202-20) were obtained from Beaver.

**Antibodies**

Anti-Caspase7 (27155-1-AP) was purchased from Proteintech. Anti-GSDME-NT (55879S) was obtained from CST.

**Methods**

**Isolation and Functional Assessment of Thoracic Aortic Rings in Mice**

The thoracic aortic ring was isolated as previously described [1]. In brief, C57BL/6 mice were euthanized using CO_2_, and the thoracic aorta was carefully excised and placed in a Na^+^-Krebs solution that had been pre-oxygenated with 95% O_2_ and 5% CO_2_. Subsequently, the vascular ring was cut into a length of approximately 2mm under a stereomicroscope. Fix the vascular ring onto the vascular tension meter (DMT), adjust the baseline tension to 3mN, and allow it to stabilize for one hour. Then 60mM K^+^ and 10^-5^M ACh were subsequently introduced to evaluate vascular ring activity.

**Immunoprecipitation-Mass Spectrometry (IP-MS)**

According to the kit instructions, add 20 µL of protein lysis buffer containing 1 mM PMSF for every 1×10^5^ cells. Take 50 µL of the protein lysis buffer and mix it with 200 µL of antibody working solution at a concentration of 5 µg/mL, then incubate the mixture overnight on a shaking platform at 4°C. The next day, add 25 µL of Protein A/G coated magnetic beads to the mixture and incubate on a shaking platform at room temperature for 15 minutes. Perform magnetic separation to isolate the beads and wash them twice with 200 µL of washing solution. Next, add 25 µL of 1x SDS-PAGE loading buffer to the washed beads, heat at 95°C for 5 minutes, and use magnetic separation to remove the beads, collecting the supernatant for SDS-PAGE or Western blot detection. For the target band in SDS-PAGE, recover it for mass spectrometry analysis, which will be performed by iProteome, Shanghai, China.

**Effects of MGO on Vascular Ring Function**

Vascular rings exhibiting optimal reactivity and functionality were carefully selected from the same mouse and divided into two groups: Control group and MGO group. The vascular rings were exposed to vehicle or 0.8mM MGO for a duration of 20 minutes, respectively, followed by stimulation with 10^-5^M Phe to induce vascular ring contraction for approximately 15 minutes. Subsequently, the tension of the vascular ring was recorded upon incremental addition of ACh (10^-9^-10^-5^ M) to assess the activity of endothelial cells. Following this evaluation, Na^+^-Krebs solution was administered three times with an interval of 2 minutes between each application. After thorough washing, another round of stimulation with 10^-5^ M Phe was performed to elicit vascular contraction for approximately 15 minutes. Finally, the tension in the vascular ring was recorded while increasing concentrations of SNP (10^-9^-10^-5^ M) were introduced to evaluate the activity of smooth muscle cells within the vasculature.

**Evaluation of Cardiac Function in Mice**

In this study, mice were anesthetized using isoflurane and cardiac function was assessed utilizing high-resolution ultrasound (Fujifilm Visual Sonics, Vevo 3100). The evaluated parameters included ejection fraction (EF), fractional shortening (FS), left ventricular anterior wall thickness (LVAW), and left ventricular posterior wall thickness (LVPW).

**Supplementary Figures**


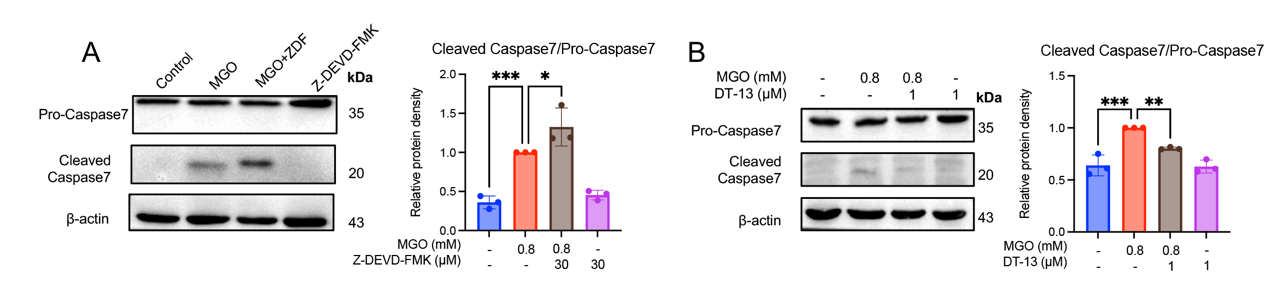


**Figure S1. Effects of Z-DEVD-FMK and DT-13 on MGO-induced Cleaved Caspase7 expression.**

**A:** HUVECs were pretreated with Z-DEVD-FMK (30μM) for 2 hours and then exposed to MGO for 24 hours. The expressions of Pro-Caspase7 and Cleaved Caspase7 were detected and analyzed by Western Blotting. **B:** HUVECs were pretreated with DT-13 (1μM) for 6 hours and then exposed to MGO for 24 hours. The expression of Pro-Caspase7 and Cleaved Caspase7 was detected by Western Blotting. (Statistical analysis was performed using two-way ANOVA, followed by Sidak test, n=3) *P<0.05, **P<0.01, ***P<0.001.


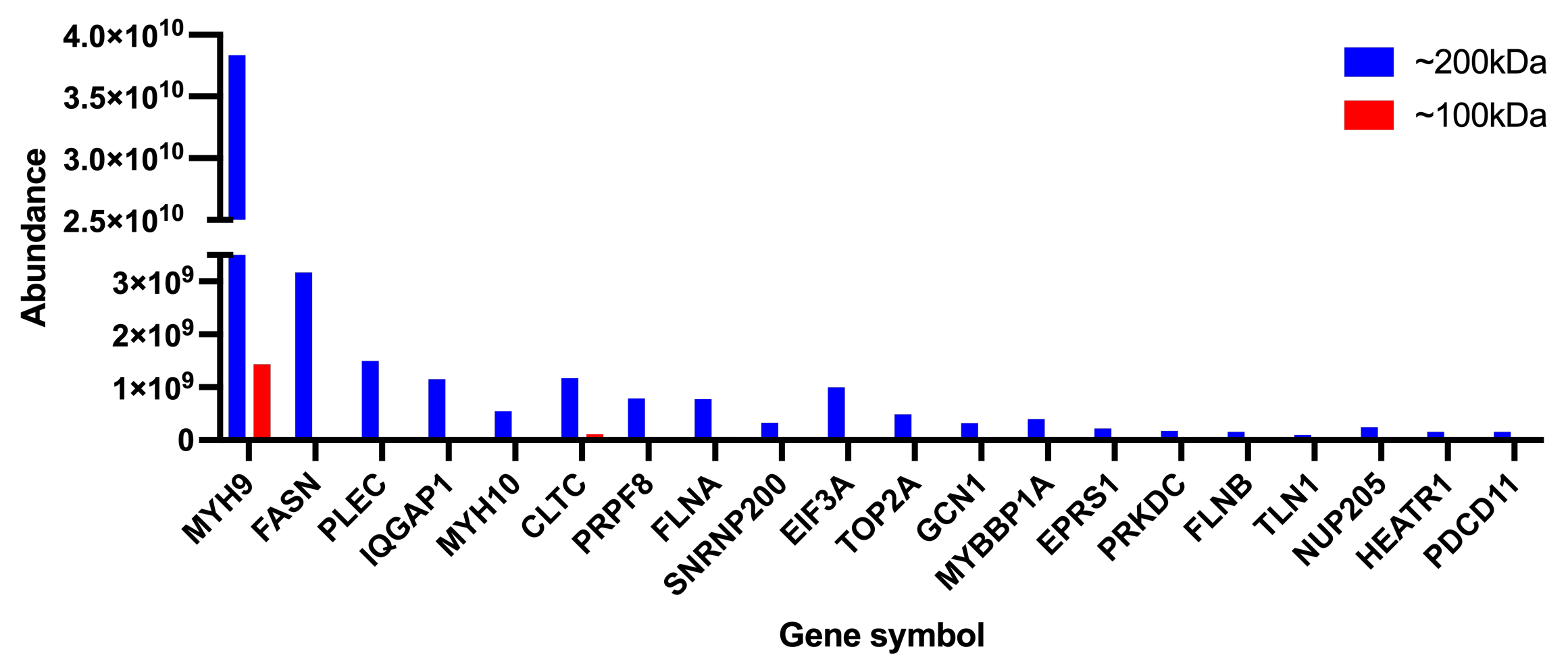


**Figure S2. Mass spectrometry analysis of protein bands cleaved by MGO in HUVECs**

Initially, immunoprecipitation (IP) technology is employed to isolate the NMMHC IIA antibody-bound protein. Subsequently, polyacrylamide gel electrophoresis is utilized to separate the protein bands, and specifically, bands around 200kDa and 100kDa are cut of and recovered through gel extraction for subsequent mass spectrometry analysis. The horizontal axis of this graph represents gene (protein) names, while the vertical axis indicates abundance of peptide segments.


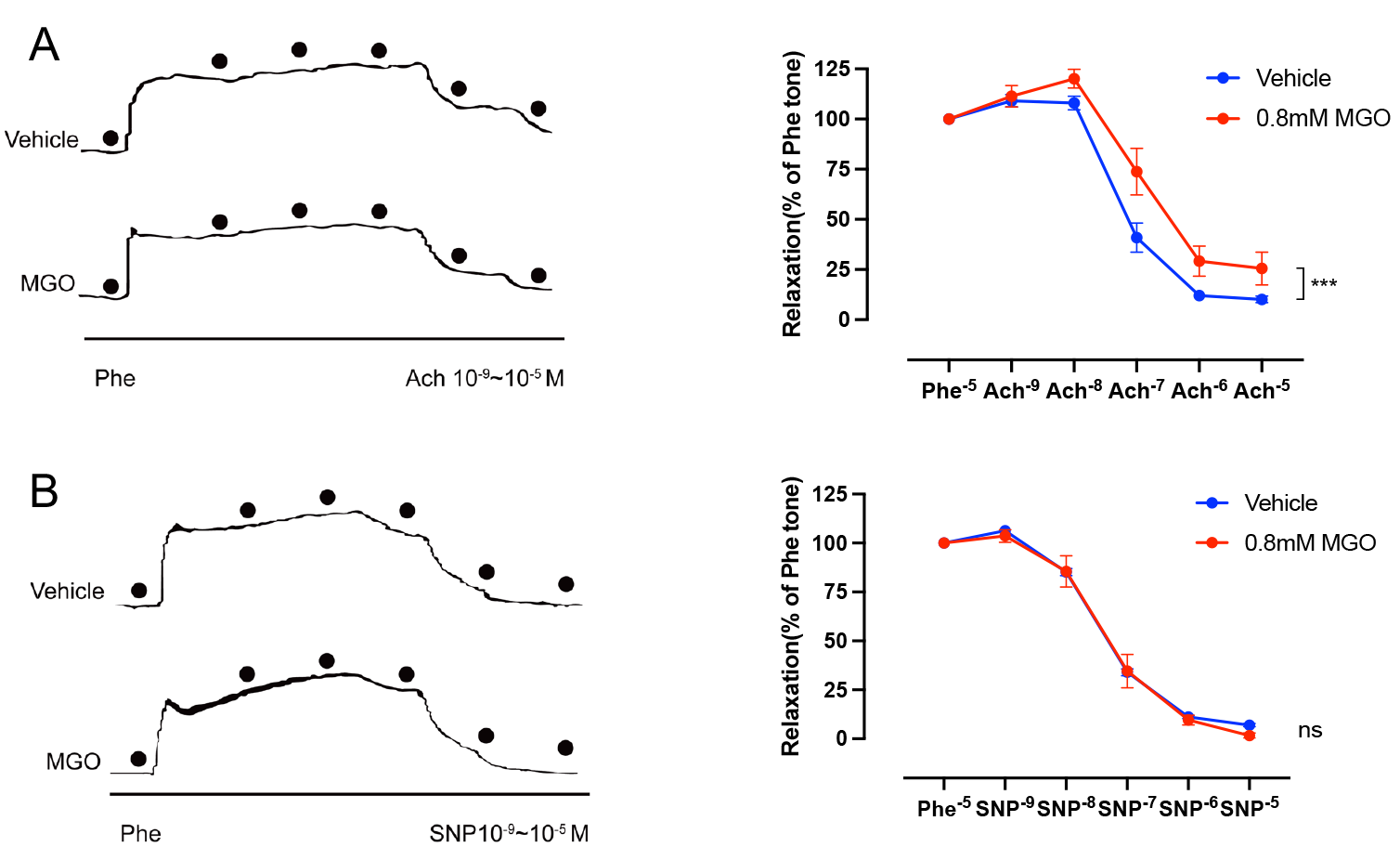


**Figure S3. The Impact of MGO on the Vascular Tone of Isolated Mesenteric Arteries in Mice.**

**A:** Following a 30-minute treatment with 0.8mM MGO or solvent in the mesenteric artery of mice, the addition of Phe (10^-5^M) to induce an increase in vascular tone, followed by a graded concentration of ACh (10^-9^-10^-5^ M) to induce a decrease in vascular tone. The left side depicts the representative diagram of vascular tone, while the right side presents the statistical analysis results. **B:** After subjecting the mesenteric artery of mice to a 30-minute treatment with 0.8mM MGO or solvent, Phe (10^-5^M) was added to provoke an elevation in vascular tone, which was then followed by a graded concentration of SNP (10^-9^-10^-5^ M) to induce a decrease in vascular tone. This sequence led to a subsequent reduction in vascular tone. The left side illustrates the representative diagram of vascular tone, and the right side are presented the corresponding statistical analysis results. The changes in vascular tension were recorded and subjected to statistical analysis, with the maximum tension induced by Phe (10^-5^M) serving as the baseline value for statistical comparison. (Statistical analysis was performed using one-way ANOVA, followed by Dunnett test, n=3) ***P<0.001.


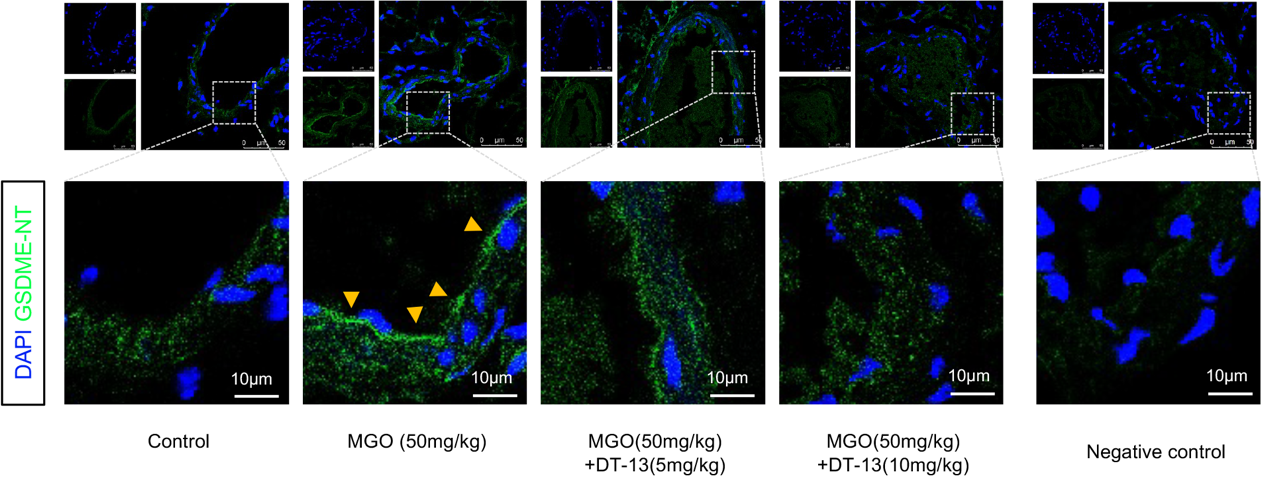


**Figure S4. DT-13 suppresses MGO-induced expression of GSDME-NT in mouse mesenteric vessels.**

The figure depicts immunostaining results of GSDME-NT in mesenteric vessels from each group of mice. Green represents GSDME-NT staining, and blue represents DAPI staining. Scale bar=10μm. (Note: The GSDME-NT antibody used here is from CST: 55879S, In the negative control group, the primary antibody against GSDME-NT was not used.)


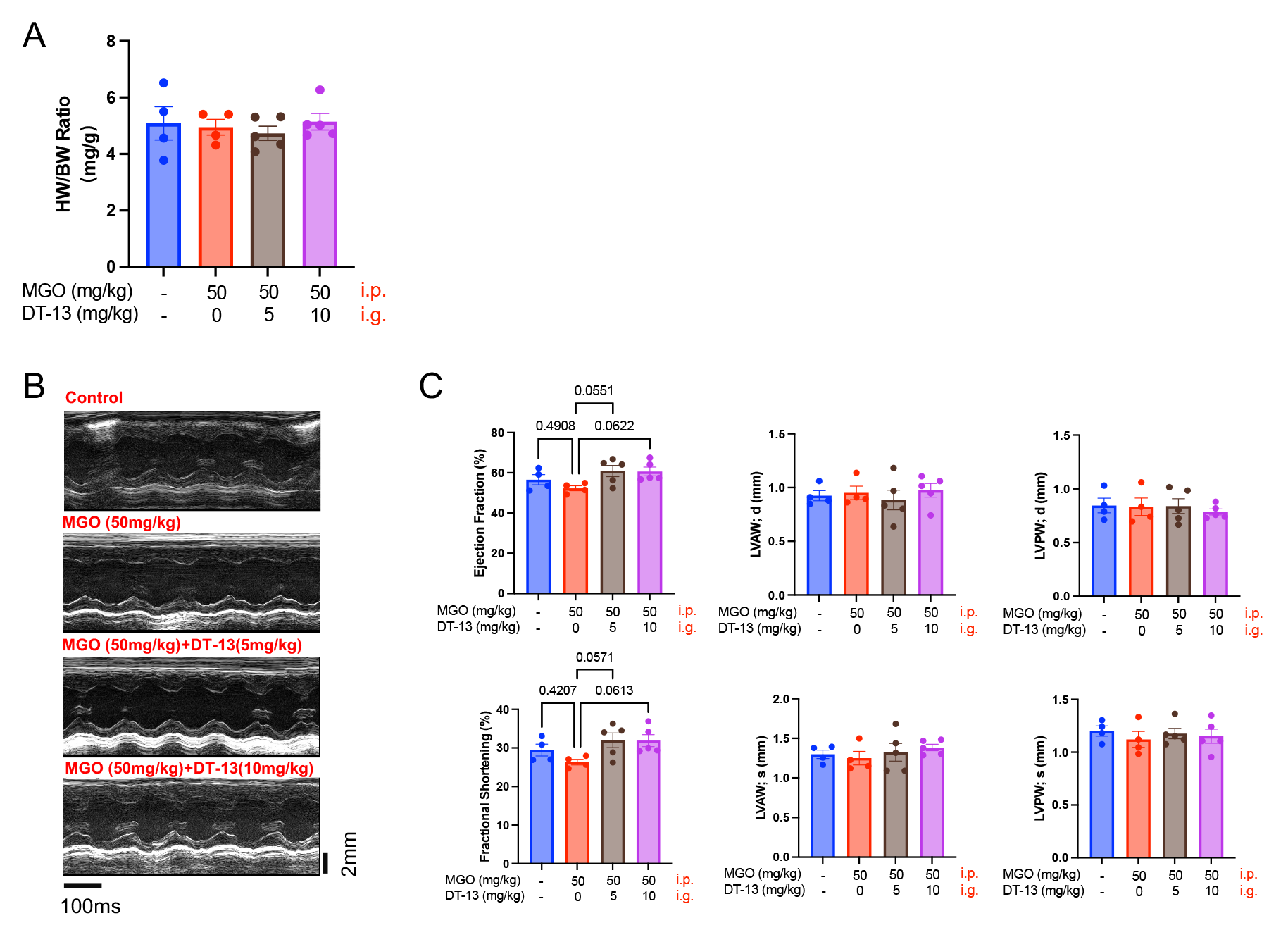


**Figure S5. No significant difference in cardiac function between MGO model group and DT-13 treatment group.**

**A:** Statistical results of heart weight to body weight ratio (HW/BW) of mice in each group after the experiment. (Statistical analysis was performed using one-way ANOVA, followed by Dunnett test, n=4-5). **B:** Echocardiography of cardiac function of mice in each group before execution. Scale bar = 100 ms (horizontal axis)/2 mm (vertical axis). **C:** Statistical analysis results of Figure B. EF refers to left ventricular ejection fraction, FS refers to left ventricular shortening fraction, and LVAW; d refers to left ventricular end-diastolic anterior wall thickness, and LVAW; s refers to left ventricular end-systolic anterior wall thickness, and LVPW; d refers to left ventricular end-diastolic posterior wall thickness, and LVPW; s refers to left ventricular end-systolic posterior wall thickness. (Statistical analysis was performed using one-way ANOVA, followed by Dunnett test, n=4-5) *P<0.05, **P<0.01, ***P<0.001.

**Supplementary Tables**

Table S1. Peptides of MYH9_result (~200KDa)

| **No.** | **Gene_symbol** | **Sequence** | **Abundance** | **RT (min)** |
| --- | --- | --- | --- | --- |
| 1 | MYH9 | MQLAKKEEELQAALAR | 7869515.625 | 22.0719 |
| 2 | MYH9 | MQQNIQELEEQLEEEESAR | 133212080 | 44.7193 |
| 3 | MYH9 | MQQNIQELEEQLEEEESAR | 26728861 | 41.412 |
| 4 | MYH9 | NAEQYKDQADK | 576874.625 | 8.9432 |
| 5 | MYH9 | NAEQYKDQADKASTR | 16537883.5 | 9.8021 |
| 6 | MYH9 | NFINNPLAQADWAAK | 318258875 | 47.0861 |
| 7 | MYH9 | NFINNPLAQADWAAKK | 14499708.25 | 37.7765 |
| 8 | MYH9 | LTKDFSALESQLQDTQELLQEENR | 1031614.625 | 55.4166 |
| 9 | MYH9 | LTEMETLQSQLMAEK | 34819502 | 21.3196 |
| 10 | MYH9 | LTEMETLQSQLMAEK | 502857526 | 41.6498 |
| 11 | MYH9 | LTEMETLQSQLMAEK | 175782722.6 | 27.4174 |
| 12 | MYH9 | QAQQERDELADEIANSSGK | 127740838.3 | 17.2694 |
| 13 | MYH9 | QEEEMMAKEEELVK | 120244960 | 18.9933 |
| 14 | MYH9 | QEEEMMAKEEELVK | 28007760 | 15.9763 |
| 15 | MYH9 | QEEEMMAKEEELVK | 6125787 | 13.5138 |
| 16 | MYH9 | NTDQASMPDNTAAQK | 3497718.438 | 9.2883 |
| 17 | MYH9 | QKHSQAVEELAEQLEQTK | 18513239.5 | 25.8292 |
| 18 | MYH9 | QKHSQAVEELAEQLEQTKR | 113349691.3 | 22.0275 |
| 19 | MYH9 | QIATLHAQVADMK | 490554224 | 17.8204 |
| 20 | MYH9 | QIATLHAQVADMK | 184147368 | 15.3013 |
| 21 | MYH9 | QIATLHAQVADMKK | 172439636 | 15.6065 |
| 22 | MYH9 | QIATLHAQVADMKK | 138880816 | 13.4287 |
| 23 | MYH9 | QLEEAEEEAQR | 195574791 | 12.6294 |
| 24 | MYH9 | QLEEAEEEAQRANASR | - | 14.1242 |
| 25 | MYH9 | NLPIYSEEIVEMYK | 346366804 | 52.9889 |
| 26 | MYH9 | NLPIYSEEIVEMYK | 122628902.9 | 43.4263 |
| 27 | MYH9 | NKHEAMITDLEER | 66739868 | 15.9065 |
| 28 | MYH9 | NKHEAMITDLEER | 39732127.5 | 13.8121 |
| 29 | MYH9 | NSFREQLEEEEEAKHNLEK | 45354579 | 16.5778 |
| 30 | MYH9 | NMDPLNDNIATLLHQSSDK | 179714034 | 47.2576 |
| 31 | MYH9 | NMDPLNDNIATLLHQSSDK | 76126952.5 | 41.6233 |
| 32 | MYH9 | NMDPLNDNIATLLHQSSDKFVSELWK | 297652713.4 | 68.9045 |
| 33 | MYH9 | NMDPLNDNIATLLHQSSDKFVSELWK | 154754524 | 68.3237 |
| 34 | MYH9 | NMDPLNDNIATLLHQSSDKFVSELWKDVDR | 9758878.75 | 69.4929 |
| 35 | MYH9 | NMDPLNDNIATLLHQSSDKFVSELWKDVDR | 2287592 | 69.0012 |
| 36 | MYH9 | LKNKHEAMITDLEER | 66607312 | 15.0856 |
| 37 | MYH9 | LKNKHEAMITDLEER | 9545211.25 | 13.0824 |
| 38 | MYH9 | LKQVEDEKNSFR | 319607768 | 11.7709 |
| 39 | MYH9 | LKSMEAEMIQLQEELAAAER | 1686558.75 | 59.8417 |
| 40 | MYH9 | LKSMEAEMIQLQEELAAAER | 3967193 | 44.8804 |
| 41 | MYH9 | LKSMEAEMIQLQEELAAAER | 5592617 | 31.7363 |
| 42 | MYH9 | LKDVLLQVDDER | 61138708 | 24.4278 |
| 43 | MYH9 | LKDVLLQVDDERR | 648069528 | 19.8844 |
| 44 | MYH9 | IIGLDQVAGMSETALPGAFK | 490019952.7 | 65.0766 |
| 45 | MYH9 | IIGLDQVAGMSETALPGAFK | 249127800.7 | 53.9017 |
| 46 | MYH9 | LEGDSTDLSDQIAELQAQIAELK | - | 69.6126 |
| 47 | MYH9 | LRLEVNLQAMK | 58615452 | 23.1554 |
| 48 | MYH9 | LQQELDDLLVDLDHQR | 524056020.3 | 55.1441 |
| 49 | MYH9 | LQVELDNVTGLLSQSDSK | 528115810.5 | 53.6716 |
| 50 | MYH9 | IRELESQISELQEDLESER | 131554342 | 45.6254 |
| 51 | MYH9 | LRLEVNLQAMK | - | 32.457 |
| 52 | MYH9 | IMGIPEEEQMGLLR | 265900904.8 | 39.7741 |
| 53 | MYH9 | IMGIPEEEQMGLLR | 515591668 | 48.0046 |
| 54 | MYH9 | VEAQLQELQVK | 194074256 | 23.1223 |
| 55 | MYH9 | VEEEAAQKNMALK | 805446.0625 | 13.4336 |
| 56 | MYH9 | VEEEEERCQHLQAEK | 482167.3125 | 11.3909 |
| 57 | MYH9 | VIQYLAYVASSHK | 991709237.9 | 30.8309 |
| 58 | MYH9 | VKLQEMEGTVK | 341630000 | 15.8695 |
| 59 | MYH9 | TVGQLYKEQLAK | 159236216 | 16.2213 |
| 60 | MYH9 | TRLQQELDDLLVDLDHQR | 49454210.88 | 61.9203 |
| 61 | MYH9 | VAAYDKLEK | 340929216 | 13.3509 |
| 62 | MYH9 | VISGVLQLGNIVFK | 259482175 | 67.0522 |
| 63 | MYH9 | YKASITALEAK | 128381300 | 16.919 |
| 64 | MYH9 | YLYVDKNFINNPLAQADWAAK | 146006098 | 53.4926 |
| 65 | MYH9 | YLYVDKNFINNPLAQADWAAKK | 6750497.25 | 46.2598 |
| 66 | MYH9 | VISGVLQLGNIVFKK | 811898144 | 55.2824 |
| 67 | MYH9 | VRTELADKVTK | 147713440 | 11.8517 |
| 68 | MYH9 | VSHLLGINVTDFTR | 971362070.8 | 41.8981 |
| 69 | MYH9 | SGFEPASLKEEVGEEAIVELVENGK | 12025212 | 61.6111 |
| 70 | MYH9 | SGFEPASLKEEVGEEAIVELVENGKK | 142442257 | 54.3147 |
| 71 | MYH9 | RALEQQVEEMK | 10735801 | 14.9936 |
| 72 | MYH9 | RALEQQVEEMKTQLEELEDELQATEDAK | 2963340.5 | 68.0611 |
| 73 | MYH9 | RDLGEELEALKTELEDTLDSTAAQQELR | - | 70.0839 |
| 74 | MYH9 | REQEVNILKK | 8035324.5 | 12.8066 |
| 75 | MYH9 | RHEMPPHIYAITDTAYR | 71344846.5 | 19.4426 |
| 76 | MYH9 | RHEMPPHIYAITDTAYR | 24090145.75 | 17.1271 |
| 77 | MYH9 | RKLEGDSTDLSDQIAELQAQIAELK | 117392617.3 | 61.6865 |
| 78 | MYH9 | QRYEILTPNSIPK | 128556936 | 25.2883 |
| 79 | MYH9 | QTLENERGELANEVK | 193173600 | 16.0719 |
| 80 | MYH9 | QVEDEKNSFR | 1587458.781 | 10.7682 |
| 81 | MYH9 | RQAQQERDELADEIANSSGK | 35449296 | 15.8413 |
| 82 | MYH9 | RQLEEAEEEAQR | 61454128 | 11.3993 |
| 83 | MYH9 | SMEAEMIQLQEELAAAER | 282074544 | 65.017 |
| 84 | MYH9 | SMEAEMIQLQEELAAAER | 37970215.06 | 36.2897 |
| 85 | MYH9 | THEAQIQEMR | 66240970 | 12.164 |
| 86 | MYH9 | TLEEEAKTHEAQIQEMR | 118281527 | 17.2469 |
| 87 | MYH9 | TEMEDLMSSKDDVGK | 37121956.5 | 11.7743 |
| 88 | MYH9 | TFHIFYYLLSGAGEHLK | 204534736.4 | 59.9512 |
| 89 | MYH9 | TFHIFYYLLSGAGEHLKTDLLLEPYNK | 10279449.5 | 69.3197 |
| 90 | MYH9 | TEMEDLMSSKDDVGK | 39487056 | 14.7853 |
| 91 | MYH9 | TLEEEAKTHEAQIQEMR | 25809841.75 | 15.3101 |
| 92 | MYH9 | SMEAEMIQLQEELAAAER | 53502710.88 | 50.0957 |
| 93 | MYH9 | TEMEDLMSSK | 22626608 | 14.349 |
| 94 | MYH9 | TEMEDLMSSK | 149131456 | 19.956 |
| 95 | MYH9 | TDLLLEPYNK | 604153408 | 31.2507 |
| 96 | MYH9 | TDLLLEPYNKYR | 43749538 | 27.9458 |
| 97 | MYH9 | TELADKVTK | 5292655 | 11.7309 |
| 98 | MYH9 | TELEDTLDSTAAQQELR | 9098247.25 | 29.7289 |
| 99 | MYH9 | EQADFAIEALAK | 622264198 | 38.523 |
| 100 | MYH9 | EQLEEEEEAKHNLEK | 226003800 | 13.6529 |
| 101 | MYH9 | ANLQIDQINTDLNLER | 336101031.9 | 42.8095 |
| 102 | MYH9 | DELADEIANSSGK | 28444590 | 19.6681 |
| 103 | MYH9 | DFSALESQLQDTQELLQEENRQK | 30383543.19 | 58.6339 |
| 104 | MYH9 | ERNTDQASMPDNTAAQK | 8277823 | 10.9748 |
| 105 | MYH9 | DFSALESQLQDTQELLQEENR | 298670844.1 | 65.9159 |
| 106 | MYH9 | DLGEELEALK | 15428132 | 39.4921 |
| 107 | MYH9 | ALELDSNLYR | 864697344 | 28.5709 |
| 108 | MYH9 | ALEQQVEEMK | 316773152 | 17.0369 |
| 109 | MYH9 | ALEQQVEEMKTQLEELEDELQATEDAK | 22853987.25 | 56.5532 |
| 110 | MYH9 | ALEQQVEEMKTQLEELEDELQATEDAKLR | 1659327.75 | 69.4202 |
| 111 | MYH9 | DLGEELEALKTELEDTLDSTAAQQELR | 24558842.25 | 72.4766 |
| 112 | MYH9 | ALEQQVEEMKTQLEELEDELQATEDAK | 89186782 | 69.6422 |
| 113 | MYH9 | EMEAELEDERK | 160642104 | 13.7712 |
| 114 | MYH9 | EMEAELEDERK | 2101215.719 | 10.9681 |
| 115 | MYH9 | EMEAELEDERKQR | 20945868.13 | 12.5582 |
| 116 | MYH9 | EMEAELEDER | 85959240 | 15.9595 |
| 117 | MYH9 | DLEGLSQRHEEK | 39584268.5 | 12.5062 |
| 118 | MYH9 | FLSNGHVTIPGQQDKDMFQETMEAMR | 24849444 | 26.7903 |
| 119 | MYH9 | FLSNGHVTIPGQQDKDMFQETMEAMR | 41658351.5 | 35.5079 |
| 120 | MYH9 | FLSNGHVTIPGQQDKDMFQETMEAMR | 77353216 | 44.3758 |
| 121 | MYH9 | FLSNGHVTIPGQQDK | 9948316.75 | 19.5176 |
| 122 | MYH9 | AQQAADKYLYVDK | 356202336 | 26.0962 |
| 123 | MYH9 | AQQAADKYLYVDKNFINNPLAQADWAAK | 147683014 | 59.0975 |
| 124 | MYH9 | AQTKEQADFAIEALAK | - | 28.9436 |
| 125 | MYH9 | FVSELWKDVDR | 16629333 | 32.2838 |
| 126 | MYH9 | ASREEILAQAK | 957201024 | 13.6413 |
| 127 | MYH9 | ASREEILAQAKENEK | 15470394.5 | 13.0669 |
| 128 | MYH9 | EEILAQAKENEK | 4185414.75 | 13.6792 |
| 129 | MYH9 | KMQQNIQELEEQLEEEESAR | 8888747 | 34.2822 |
| 130 | MYH9 | KMQQNIQELEEQLEEEESAR | 35458141.5 | 39.5404 |
| 131 | MYH9 | KRHEMPPHIYAITDTAYR | 52496122 | 16.2479 |
| 132 | MYH9 | KRHEMPPHIYAITDTAYR | 114631535.9 | 17.9437 |
| 133 | MYH9 | KQELEEICHDLEAR | 2313912.5 | 22.9984 |
| 134 | MYH9 | EEVGEEAIVELVENGKK | 1911282.5 | 44.0561 |
| 135 | MYH9 | KGAGDGSDEEVDGKADGAEAKPAE | 2801534.25 | 10.9566 |
| 136 | MYH9 | DMFQETMEAMR | 9962245.125 | 27.2354 |
| 137 | MYH9 | DMFQETMEAMR | 19804928 | 42.1606 |
| 138 | MYH9 | KKMQQNIQELEEQLEEEESAR | 1289583.75 | 28.3809 |
| 139 | MYH9 | KIRELESQISELQEDLESER | 1455771.25 | 41.7222 |
| 140 | MYH9 | KLEGDSTDLSDQIAELQAQIAELK | 184352762.1 | 67.9353 |
| 141 | MYH9 | KKVEAQLQELQVK | 662425568 | 15.9882 |
| 142 | MYH9 | DVDRIIGLDQVAGMSETALPGAFK | 3000138.5 | 63.9394 |
| 143 | MYH9 | DVLLQVDDER | 19158944 | 25.6892 |
| 144 | MYH9 | DVLLQVDDERR | 303776481.5 | 19.9575 |
| 145 | MYH9 | IAQLEEELEEEQGNTELINDR | 198913152 | 40.3888 |
| 146 | MYH9 | IAQLEEQLDNETKER | 249190836 | 18.5672 |
| 147 | MYH9 | IAQLEEQLDNETK | 295465568 | 20.7192 |
| 148 | MYH9 | KVIQYLAYVASSHK | 224887910 | 25.1471 |
| 149 | MYH9 | KVEAQLQELQVK | 362491552 | 18.7026 |
| 150 | MYH9 | KTLEEEAKTHEAQIQEMR | 1161704.25 | 14.5831 |
| 151 | MYH9 | KTLEEEAKTHEAQIQEMR | 18321220 | 16.4305 |
| 152 | MYH9 | IAEFTTNLTEEEEKSK | 421356808 | 20.95 |
| 153 | MYH9 | IAEFTTNLTEEEEK | 258209792 | 26.0309 |
| 154 | MYH9 | HSQAVEELAEQLEQTKR | 449698286.8 | 27.5302 |
| 155 | MYH9 | HSQAVEELAEQLEQTK | 64131566 | 32.7135 |
| 156 | MYH9 | ELESQISELQEDLESER | 59615696 | 47.6963 |
| 157 | MYH9 | AKQTLENERGELANEVK | 151699476 | 14.5435 |
| 158 | MYH9 | ALEEAMEQKAELER | 53977002 | 17.9389 |
| 159 | MYH9 | HEAMITDLEER | 115833191 | 18.2677 |
| 160 | MYH9 | HEAMITDLEER | 10550851 | 15.6659 |
| 161 | MYH9 | HEMPPHIYAITDTAYR | 137754893 | 23.4539 |
| 162 | MYH9 | HEMPPHIYAITDTAYR | 95539621 | 19.834 |
| 163 | MYH9 | KAGKLDPHLVLDQLR | 6019671.75 | 27.983 |
| 164 | MYH9 | KANLQIDQINTDLNLER | 307615186 | 34.386 |
| 165 | MYH9 | EKQLAAENR | 409146.6563 | 8.8238 |
| 166 | MYH9 | EQLEEEEEAK | 513307.125 | 11.9193 |
| 167 | MYH9 | DMFQETMEAMR | 3324588.25 | 18.0616 |
| 168 | MYH9 | EMEAELEDERKQR | 2248916.938 | 10.0559 |
| 169 | MYH9 | EMEAELEDER | 22131732 | 13.0152 |
| 170 | MYH9 | THEAQIQEMR | 1853670.25 | 9.3845 |
| 171 | MYH9 | KEEELQAALARVEEEAAQK | 1359178.875 | 37.8701 |
| 172 | MYH9 | IMGIPEEEQMGLLR | 64267449.75 | 30.3069 |
| 173 | MYH9 | ALEQQVEEMK | 23771716 | 13.416 |
| 174 | MYH9 | LEVNLQAMK | 144495216 | 18.2048 |
| 175 | MYH9 | LEVNLQAMK | 496514880 | 27.3634 |
| 176 | MYH9 | KLVWVPSDK | 421157536 | 19.6063 |
| 177 | MYH9 | LQKDLEGLSQR | 2924296 | 15.2016 |
| 178 | MYH9 | ASITALEAK | 738307712 | 17.4175 |
| 179 | MYH9 | AQFERDLQGR | 969891.5 | 13.3019 |
| 180 | MYH9 | VEEEAAQKNMALKK | 8459175 | 11.4317 |
| 181 | MYH9 | LVWVPSDK | 420033088 | 25.3849 |
| 182 | MYH9 | VNKDDIQK | 1422248.875 | 8.8633 |
| 183 | MYH9 | TLEEEAK | 1497230.25 | 10.1028 |
| 184 | MYH9 | ALEEAMEQK | 326003808 | 14.2532 |
| 185 | MYH9 | YEILTPNSIPK | 385342968.3 | 32.4931 |
| 186 | MYH9 | KLQAQMKDCMR | 2053576.375 | 12.9992 |
| 187 | MYH9 | RGDLPFVVPR | 1033455924 | 29.1971 |
| 188 | MYH9 | LQEMEGTVK | 272232768 | 14.2181 |
| 189 | MYH9 | LQEMEGTVK | 2865945 | 12.4238 |
| 190 | MYH9 | ALEEAMEQK | 5413179.688 | 13.0585 |
| 191 | MYH9 | SGFEPASLK | 26878768 | 18.2728 |
| 192 | MYH9 | REQEVNILK | 48432667.5 | 15.2923 |
| 193 | MYH9 | GALALEEKR | 39588772 | 13.7702 |
| 194 | MYH9 | AQQAADK | 291455.75 | 9.3828 |
| 195 | MYH9 | VEDMAELTCLNEASVLHNLKER | 944916.9375 | 44.2455 |
| 196 | MYH9 | SVHELEK | 4398539 | 9.2967 |
| 197 | MYH9 | RQQQLTAMK | 385272.875 | 11.0019 |
| 198 | MYH9 | LRNWQWWR | 29437970 | 30.5123 |
| 199 | MYH9 | NWQWWR | 365052097.4 | 38.4234 |
| 200 | MYH9 | QACVLMIK | 5526447 | 35.2988 |
| 201 | MYH9 | EQEVNILKK | 334904160 | 14.9602 |
| 202 | MYH9 | GDLPFVVPR | 186175232 | 39.9996 |
| 203 | MYH9 | VMQEQGTHPK | 1714762.125 | 8.6591 |
| 204 | MYH9 | KKLEMDLK | 7105649.875 | 13.1281 |
| 205 | MYH9 | QQQLTAMK | 917960.375 | 9.9753 |
| 206 | MYH9 | QQQLTAMK | 5014838 | 13.419 |
| 207 | MYH9 | VKPLLQVSR | 786716608 | 17.0918 |
| 208 | MYH9 | VAAYDK | 188578.0469 | 9.3029 |
| 209 | MYH9 | VVFQEFR | 1123028224 | 24.5088 |
| 210 | MYH9 | KLKDVLLQVDDER | 7075166 | 21.511 |
| 211 | MYH9 | DQGELER | 1619833.625 | 10.3789 |
| 212 | MYH9 | FVSELWK | 230304160 | 29.6528 |
| 213 | MYH9 | KTLEEEAK | 234413.4219 | 9.2186 |

Table S2. Peptides of MYH9_result (~100KDa)

| **No.** | **Gene_symbol** | **Sequence** | **Abundance** | **RT (min)** |
| --- | --- | --- | --- | --- |
| 1 | MYH9 | RGDLPFVVPR | 112778264 | 29.3042 |
| 2 | MYH9 | VAAYDKLEK | 72107724 | 13.125 |
| 3 | MYH9 | ASITALEAK | 67612664 | 17.284 |
| 4 | MYH9 | LKDVLLQVDDERR | 52436740 | 19.5489 |
| 5 | MYH9 | KFDQLLAEEK | 44860668 | 18.4506 |
| 6 | MYH9 | ALEEAMEQK | 44052628 | 14.2533 |
| 7 | MYH9 | QIATLHAQVADMK | 39701037 | 17.8944 |
| 8 | MYH9 | HSQAVEELAEQLEQTKR | 38253548.88 | 27.7203 |
| 9 | MYH9 | KKVEAQLQELQVK | 35950350.5 | 16.0684 |
| 10 | MYH9 | LEVNLQAMK | 35007656 | 27.1076 |
| 11 | MYH9 | LQQELDDLLVDLDHQR | 34355580.31 | 54.2213 |
| 12 | MYH9 | FDQLLAEEK | 34014500 | 20.8459 |
| 13 | MYH9 | ALEQQVEEMK | 33462698 | 17.0731 |
| 14 | MYH9 | ASREEILAQAK | 31149808 | 13.6823 |
| 15 | MYH9 | VKLQEMEGTVK | 29512945 | 15.92 |
| 16 | MYH9 | LQVELDNVTGLLSQSDSK | 29466344.5 | 54.0765 |
| 17 | MYH9 | ELEDATETADAMNR | 29272926 | 16.8797 |
| 18 | MYH9 | IAQLEEQLDNETK | 27222872 | 20.8494 |
| 19 | MYH9 | EQEVNILKK | 27032908 | 15.0159 |
| 20 | MYH9 | LKQVEDEKNSFR | 25061702 | 11.7912 |
| 21 | MYH9 | KANLQIDQINTDLNLER | 24288898.5 | 34.8143 |
| 22 | MYH9 | ANLQIDQINTDLNLER | 23997230 | 42.749 |
| 23 | MYH9 | DVLLQVDDERR | 22410389 | 20.0764 |
| 24 | MYH9 | VIQYLAYVASSHK | 20778929 | 30.9083 |
| 25 | MYH9 | VSHLLGINVTDFTR | 20126869 | 42.161 |
| 26 | MYH9 | LDPHLVLDQLR | 19829698 | 41.5018 |
| 27 | MYH9 | QIATLHAQVADMKK | 17965269.5 | 15.6358 |
| 28 | MYH9 | ALELDSNLYR | 17178034 | 28.3582 |
| 29 | MYH9 | VEAQLQELQVK | 17107414 | 23.1699 |
| 30 | MYH9 | QLEEAEEEAQR | 16990200 | 12.7299 |
| 31 | MYH9 | IAQLEEQLDNETKER | 16863862.75 | 18.612 |
| 32 | MYH9 | AGVLAHLEEER | 16176291.5 | 17.9945 |
| 33 | MYH9 | VISGVLQLGNIVFKK | 16103097.5 | 55.4379 |
| 34 | MYH9 | EMEAELEDERK | 16060900.5 | 13.8073 |
| 35 | MYH9 | VRTELADKVTK | 15637567 | 11.8936 |
| 36 | MYH9 | KVEAQLQELQVK | 14807660 | 18.5479 |
| 37 | MYH9 | EQLEEEEEAKHNLEK | 13756219.5 | 13.5218 |
| 38 | MYH9 | IAQLEEELEEEQGNTELINDR | 13680200.5 | 40.6357 |
| 39 | MYH9 | TEMEDLMSSK | 13511648 | 19.8079 |
| 40 | MYH9 | QAQQERDELADEIANSSGK | 13085754.5 | 17.3649 |
| 41 | MYH9 | GALALEEKR | 12118492 | 13.7435 |
| 42 | MYH9 | LEVNLQAMK | 10981517 | 18.251 |
| 43 | MYH9 | DFSALESQLQDTQELLQEENR | 10876560.25 | 66.0107 |
| 44 | MYH9 | SMEAEMIQLQEELAAAER | 10698615 | 65.1629 |
| 45 | MYH9 | EQADFAIEALAK | 9797859 | 38.1401 |
| 46 | MYH9 | QLLQANPILEAFGNAK | 8985695.375 | 62.3107 |
| 47 | MYH9 | LVWVPSDK | 8869244 | 25.1513 |
| 48 | MYH9 | EMEAELEDER | 8844573 | 16.0067 |
| 49 | MYH9 | YEILTPNSIPK | 7964719.5 | 32.7936 |
| 50 | MYH9 | TEMEDLMSSKDDVGK | 7860012 | 19.1175 |
| 51 | MYH9 | TLEEEAKTHEAQIQEMR | 7698966 | 17.2915 |
| 52 | MYH9 | AQQAADKYLYVDK | 7105066.5 | 26.1058 |
| 53 | MYH9 | QRYEILTPNSIPK | 6598723.875 | 25.0814 |
| 54 | MYH9 | RQLEEAEEEAQR | 6354493.5 | 11.4318 |
| 55 | MYH9 | HSQAVEELAEQLEQTK | 5618015 | 32.9942 |
| 56 | MYH9 | IIGLDQVAGMSETALPGAFK | 5434081.125 | 65.3583 |
| 57 | MYH9 | NFINNPLAQADWAAK | 5226387.5 | 47.329 |
| 58 | MYH9 | DELADEIANSSGK | 5203438 | 19.7444 |
| 59 | MYH9 | VDYKADEWLMK | 4764243.625 | 27.5295 |
| 60 | MYH9 | ALEEAMEQKAELER | 4753417.75 | 17.869 |
| 61 | MYH9 | FVSELWK | 4630303.5 | 30.1437 |
| 62 | MYH9 | NLPIYSEEIVEMYK | 4270940 | 52.8754 |
| 63 | MYH9 | NTDQASMPDNTAAQK | 4253418 | 12.2694 |
| 64 | MYH9 | AGVLAHLEEERDLK | 4192864.75 | 19.138 |
| 65 | MYH9 | IIGLDQVAGMSETALPGAFK | 4090508.125 | 53.834 |
| 66 | MYH9 | TQLEELEDELQATEDAK | 4085148 | 45.7926 |
| 67 | MYH9 | YKASITALEAK | 3708482.75 | 16.9645 |
| 68 | MYH9 | VISGVLQLGNIVFK | 3572087.5 | 67.2588 |
| 69 | MYH9 | LRLEVNLQAMK | 3377610 | 32.6541 |
| 70 | MYH9 | DLEGLSQRHEEK | 3106390.75 | 12.5872 |
| 71 | MYH9 | TEMEDLMSSKDDVGK | 3030706.25 | 14.8406 |
| 72 | MYH9 | KVIQYLAYVASSHK | 3023449 | 25.3872 |
| 73 | MYH9 | HEMPPHIYAITDTAYR | 2946359.5 | 23.1471 |
| 74 | MYH9 | LQQLFNHTMFILEQEEYQR | 2864255.75 | 52.493 |
| 75 | MYH9 | TEMEDLMSSKDDVGK | 2790272 | 11.7965 |
| 76 | MYH9 | AGKLDPHLVLDQLR | 2579904.75 | 35.2494 |
| 77 | MYH9 | EMEAELEDER | 2394721.75 | 13.0663 |
| 78 | MYH9 | NMDPLNDNIATLLHQSSDK | 2282497.5 | 47.6076 |
| 79 | MYH9 | NLPIYSEEIVEMYK | 2200801 | 43.7977 |
| 80 | MYH9 | NMDPLNDNIATLLHQSSDKFVSELWK | 2158739 | 68.8529 |
| 81 | MYH9 | TEMEDLMSSK | 2061624.625 | 14.3917 |
| 82 | MYH9 | SMEAEMIQLQEELAAAER | 1790731.625 | 50.2961 |
| 83 | MYH9 | HEMPPHIYAITDTAYR | 1617644 | 20.0206 |
| 84 | MYH9 | DVLLQVDDER | 1609287.25 | 25.8801 |
| 85 | MYH9 | LQQLFNHTMFILEQEEYQR | 1598108.5 | 42.7979 |
| 86 | MYH9 | LKDVLLQVDDER | 1360679.875 | 24.539 |
| 87 | MYH9 | DFSALESQLQDTQELLQEENRQK | 1245794.625 | 58.9753 |
| 88 | MYH9 | TFHIFYYLLSGAGEHLK | 1189140.125 | 61.4835 |
| 89 | MYH9 | TRLQQELDDLLVDLDHQR | 1160140.719 | 63.6114 |
| 90 | MYH9 | ALEQQVEEMKTQLEELEDELQATEDAK | 978573.3125 | 69.5851 |
| 91 | MYH9 | SMEAEMIQLQEELAAAER | 953590.5625 | 36.5755 |
| 92 | MYH9 | SVHELEK | 659573.1875 | 9.2585 |
| 93 | MYH9 | SMAVAAR | 523953.7813 | 11.501 |
| 94 | MYH9 | EMEAELEDERK | 496942.3438 | 10.9546 |
| 95 | MYH9 | TELADKVTK | 308987.9063 | 11.7656 |
| 96 | MYH9 | THEAQIQEMR | 177162.6094 | 9.4628 |
| 97 | MYH9 | QIATLHAQVADMK | - | 15.3811 |
| 98 | MYH9 | INFDVNGYIVGANIETYLLEK | - | 69.8837 |
| 99 | MYH9 | QIATLHAQVADMKK | - | 13.5288 |
| 100 | MYH9 | AKQTLENERGELANEVK | - | 14.5976 |
| 101 | MYH9 | NAEQYKDQADKASTR | - | 9.7756 |

**References**

1. Xu H, Fang B, Du S, Wang S, Li Q, Jia X, Bao C, Ye L, Sui X, Qian L, et al: **Endothelial cell prostaglandin E2 receptor EP4 is essential for blood pressure homeostasis.** *JCI Insight* 2020, **5**.
