# Supplementary material for: Promoting the transition from pyroptosis to apoptosis in endothelial cells: a novel approach to alleviate methylglyoxal-induced vascular damage
Source: J Transl Med. 2025 Feb 10;23:170. doi: 10.1186/s12967-025-06195-x (PMC11809013; doi:10.1186/s12967-025-06195-x)
Supplement: Supplementary file 2 — Supplementary Material 2 [file 12967_2025_6195_MOESM2_ESM.doc]

**Supplementary materials**

This document is divided into three sections:

1. Original Western blot images.

2. Positive and negative controls for Western blotting.

3. Negative control for immunofluorescence staining.

1. **The original western blots**


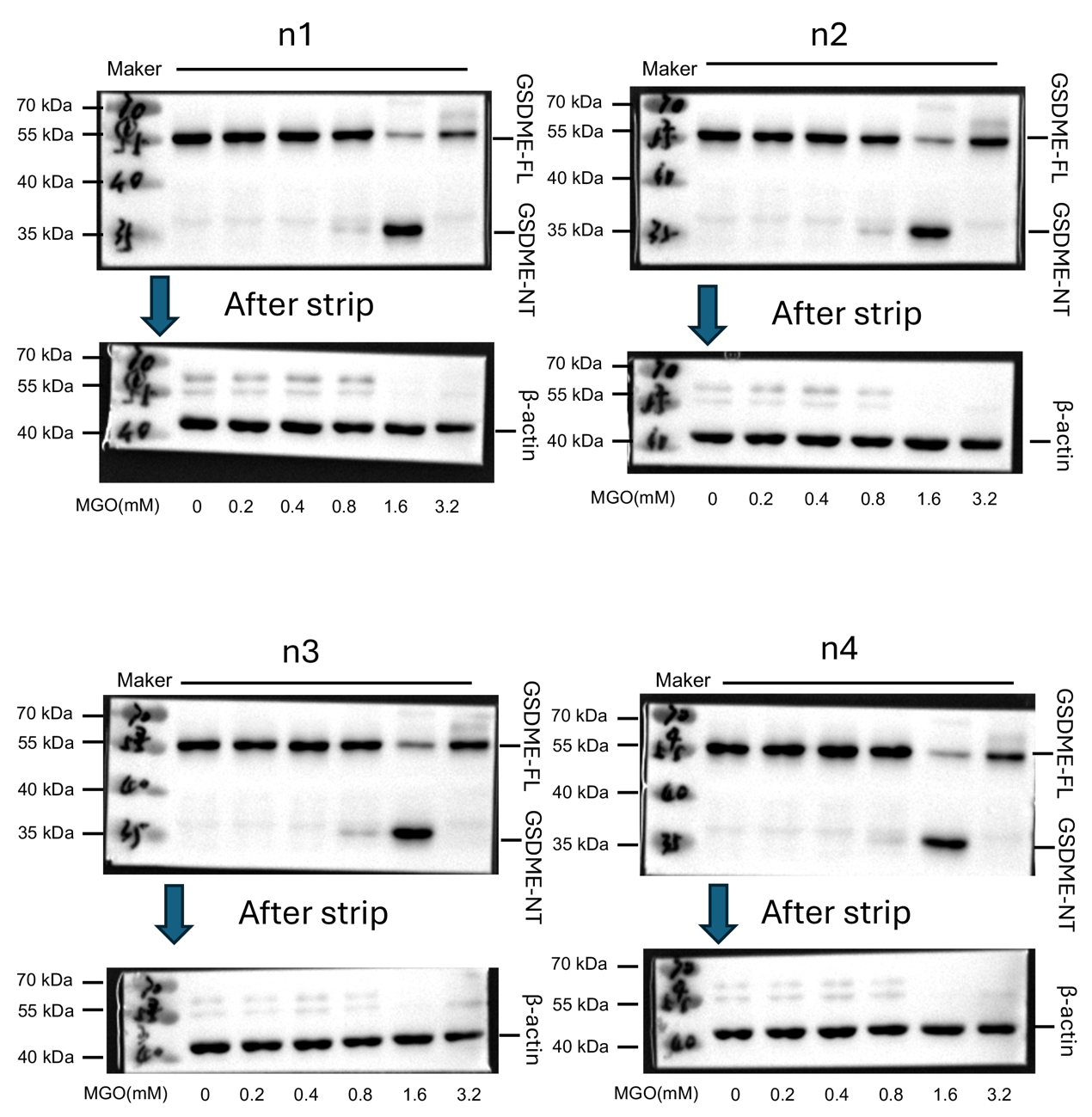


The original western blots of Figure 2 B


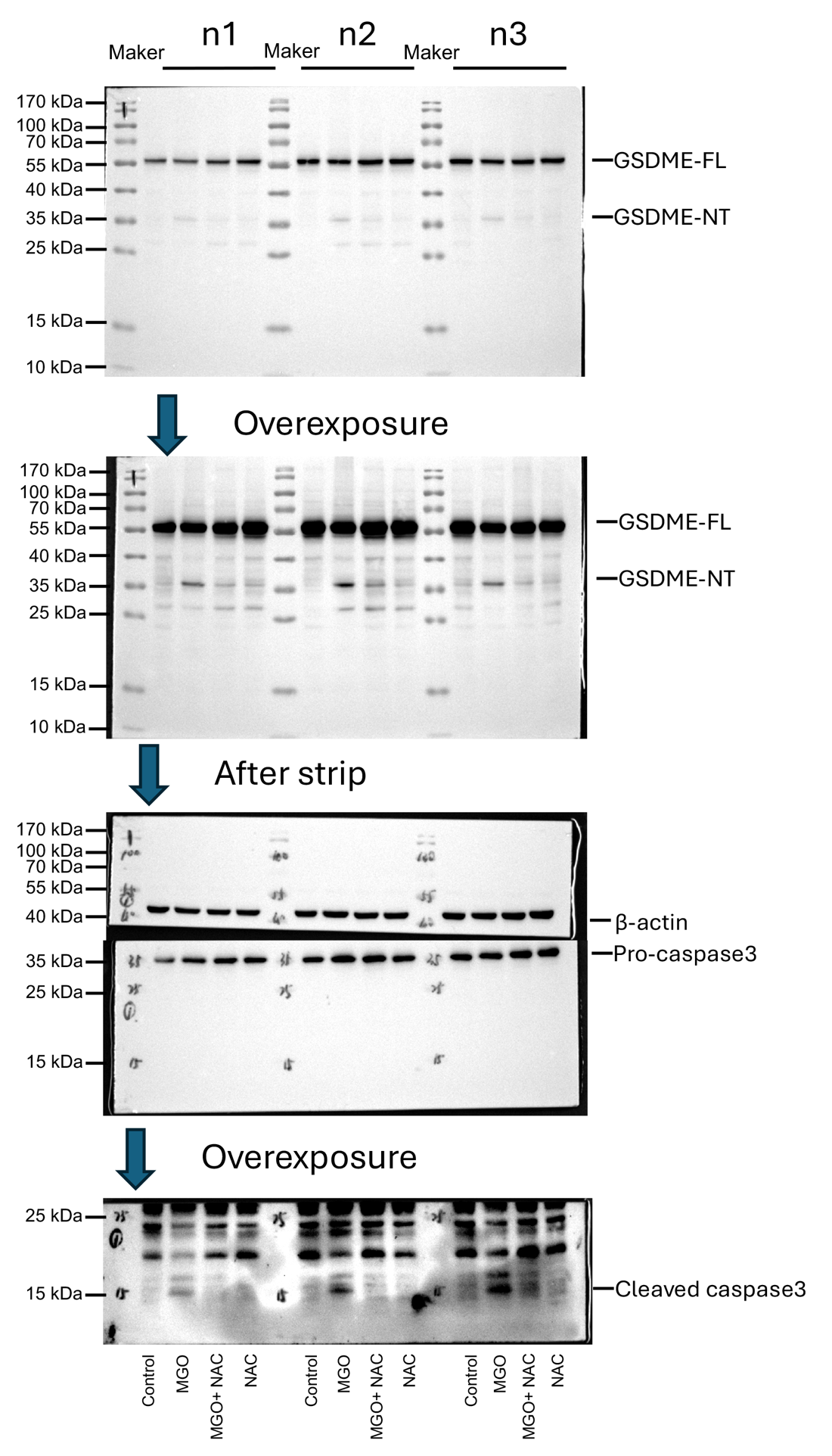


The original western blots of Figure 2 E(top)


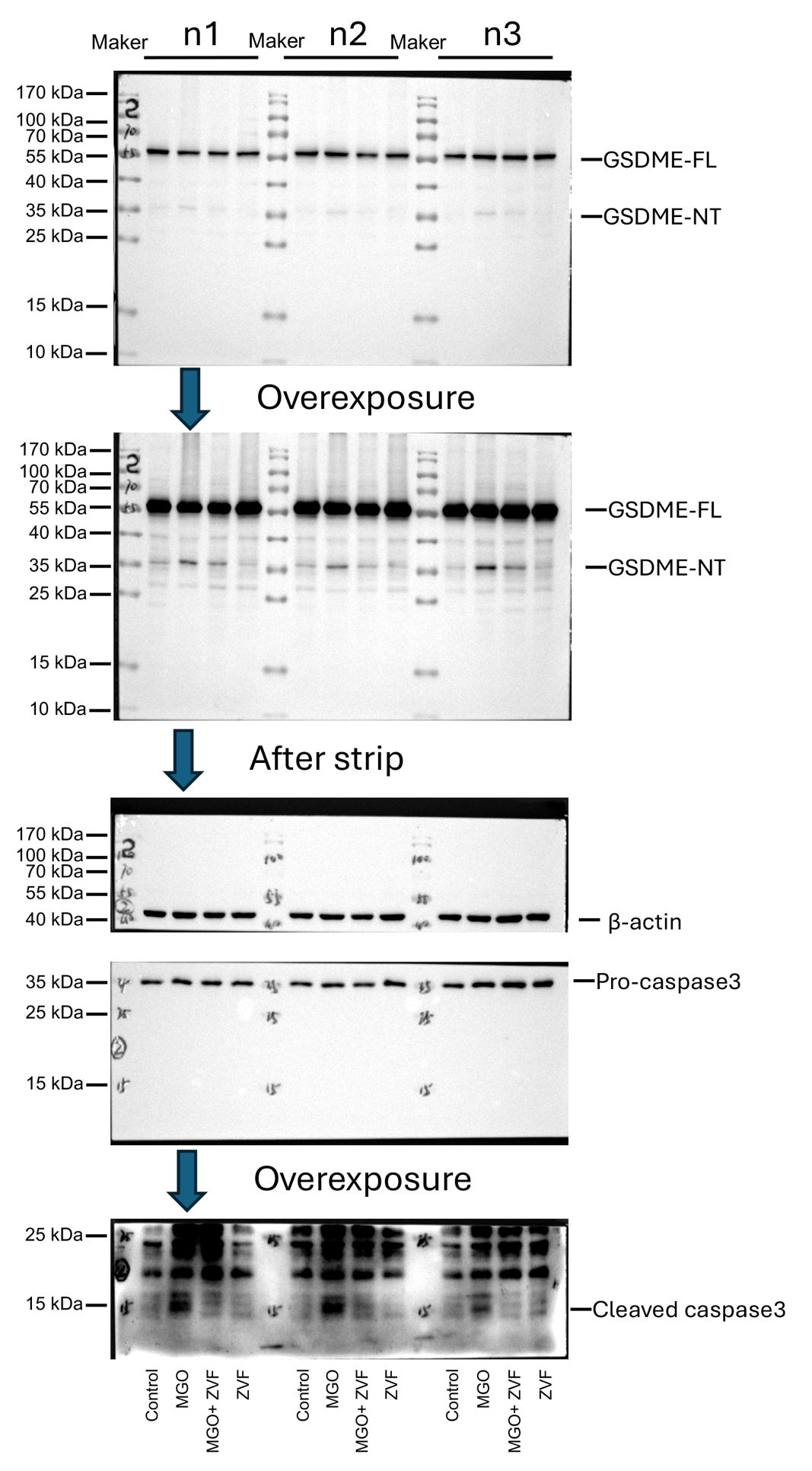


The original western blots of Figure 2 E(middle)


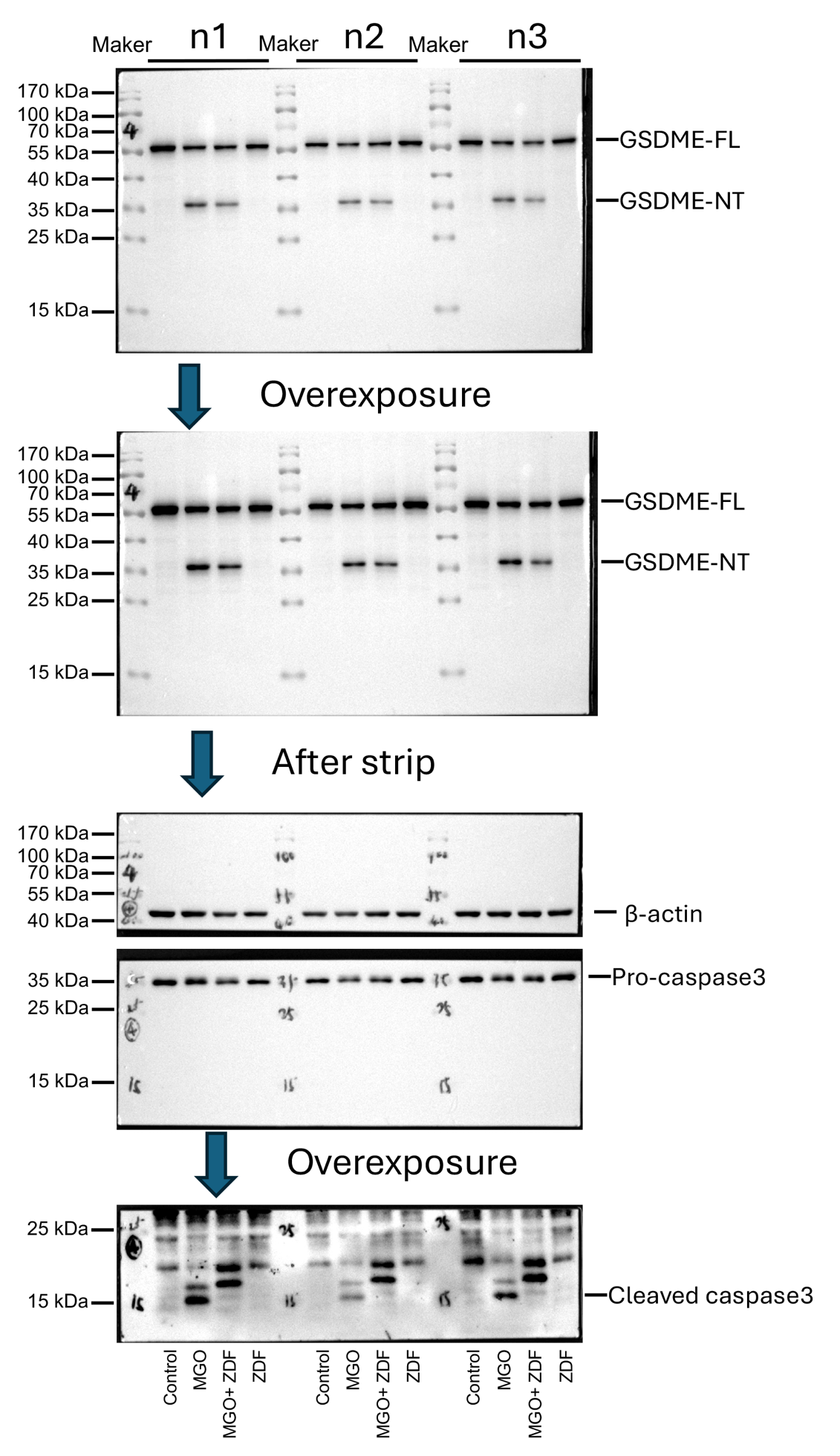


The original western blots of Figure 2 E(bottom)


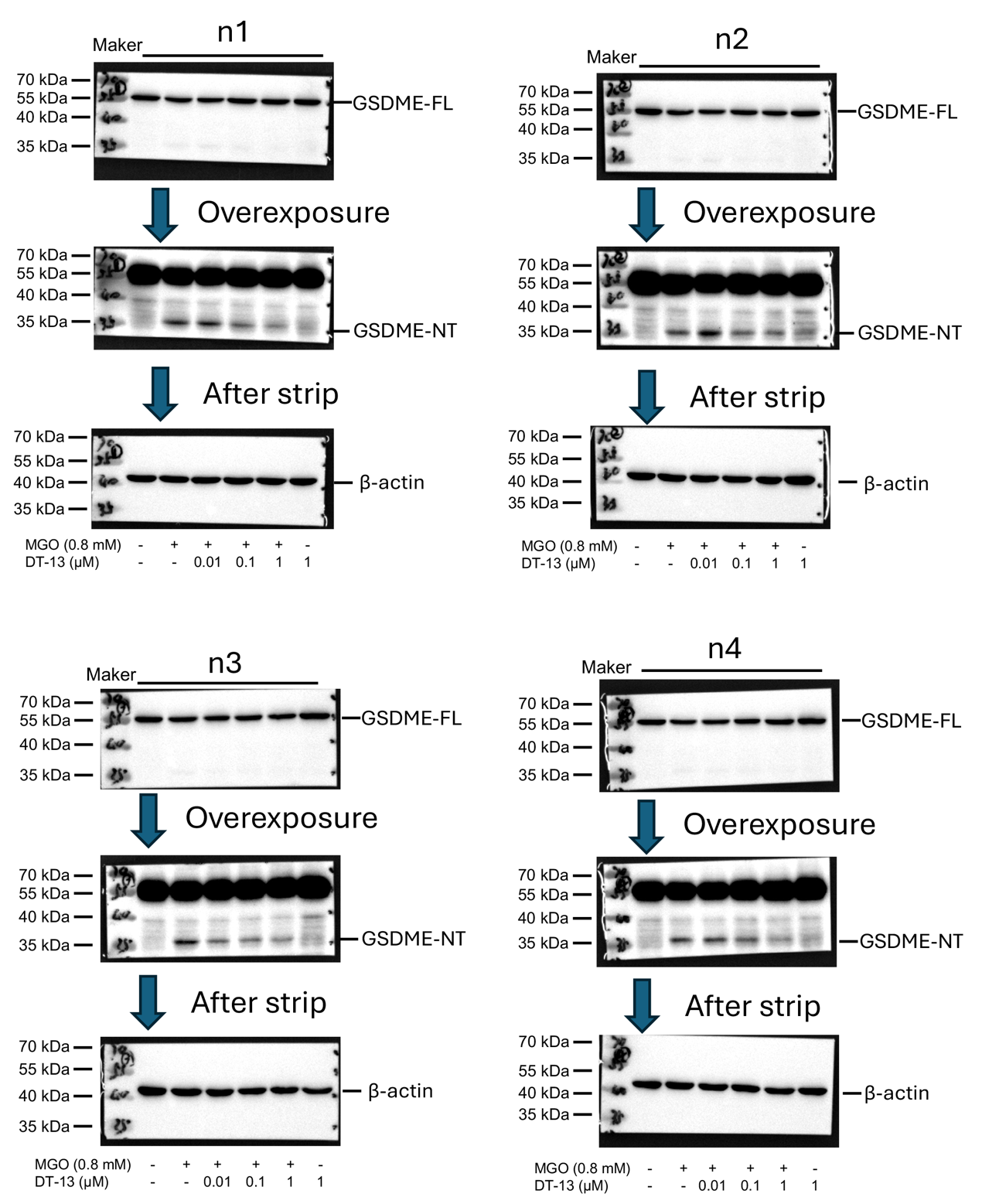


The original western blots of Figure 3D


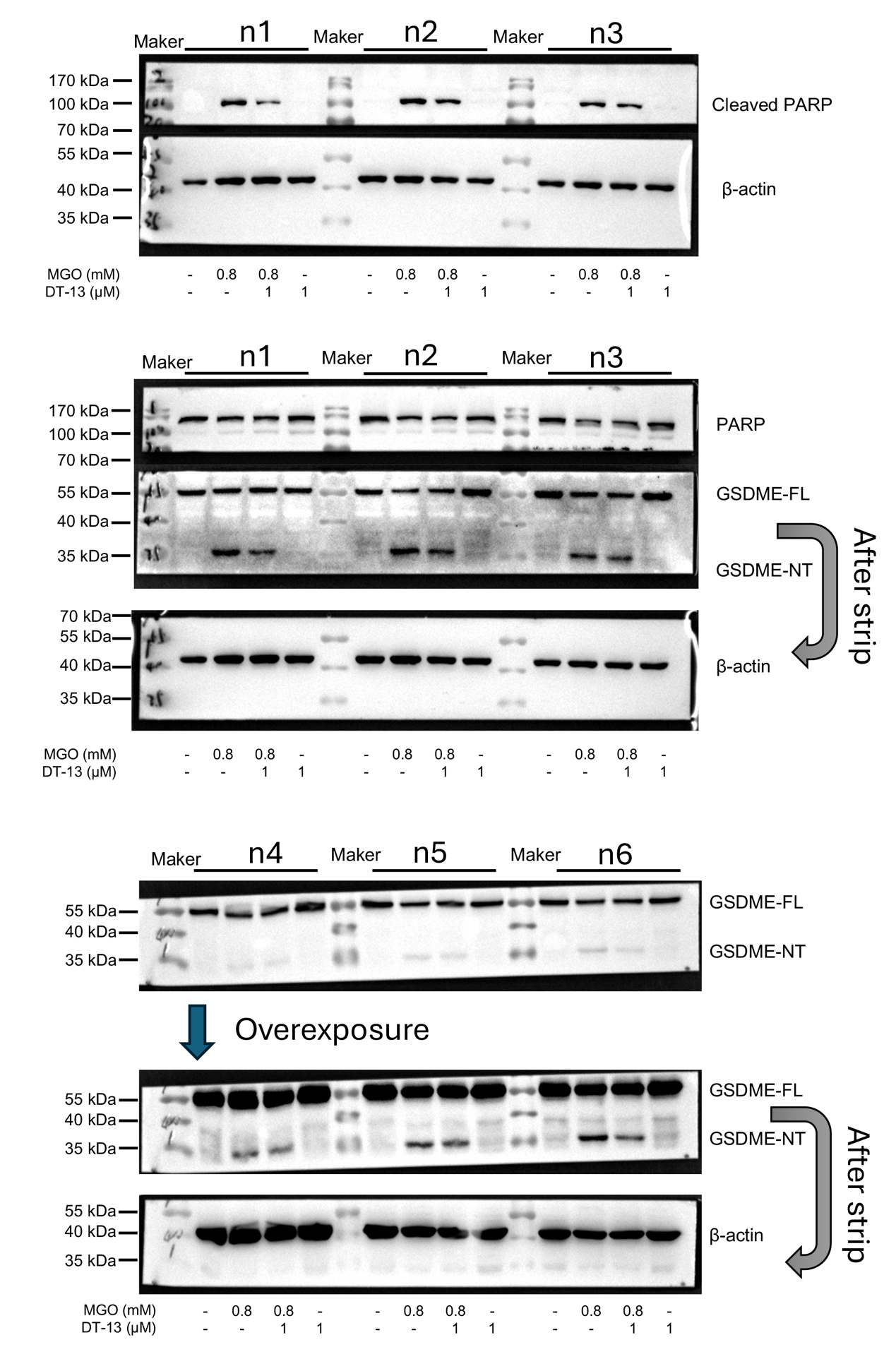


The original western blots of Figure 3E-G


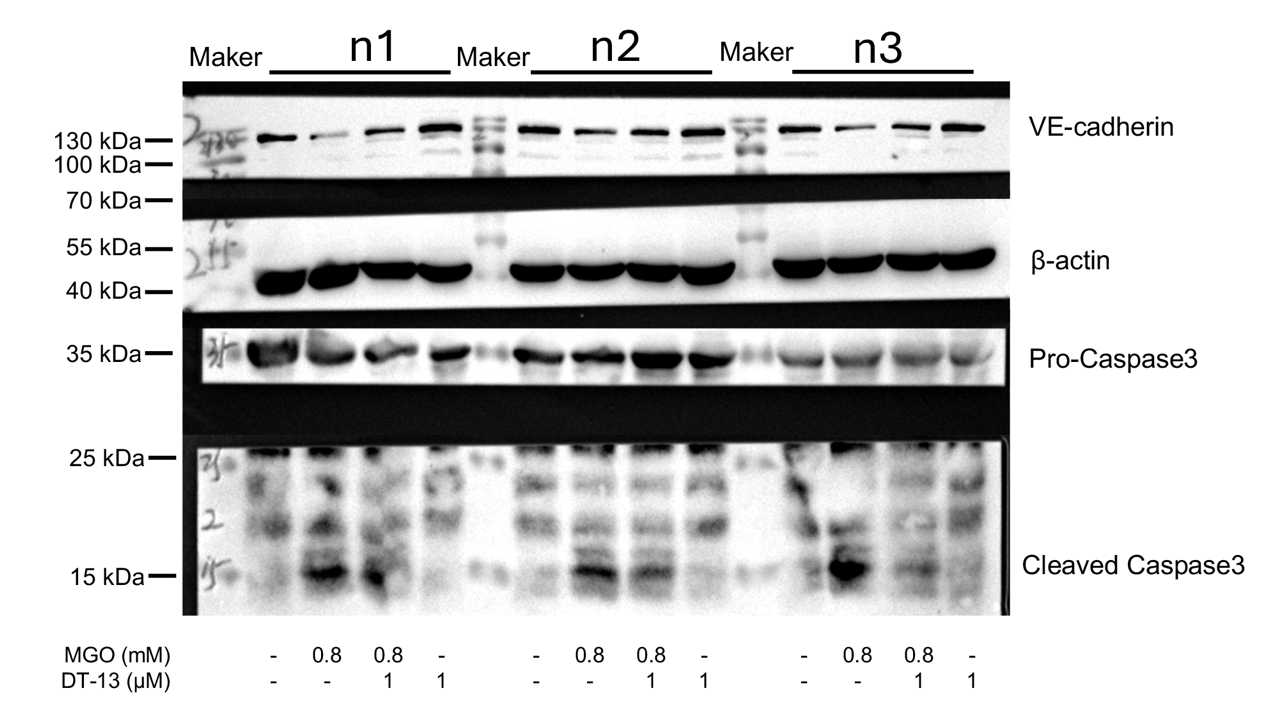


The original western blots of Figure 3H


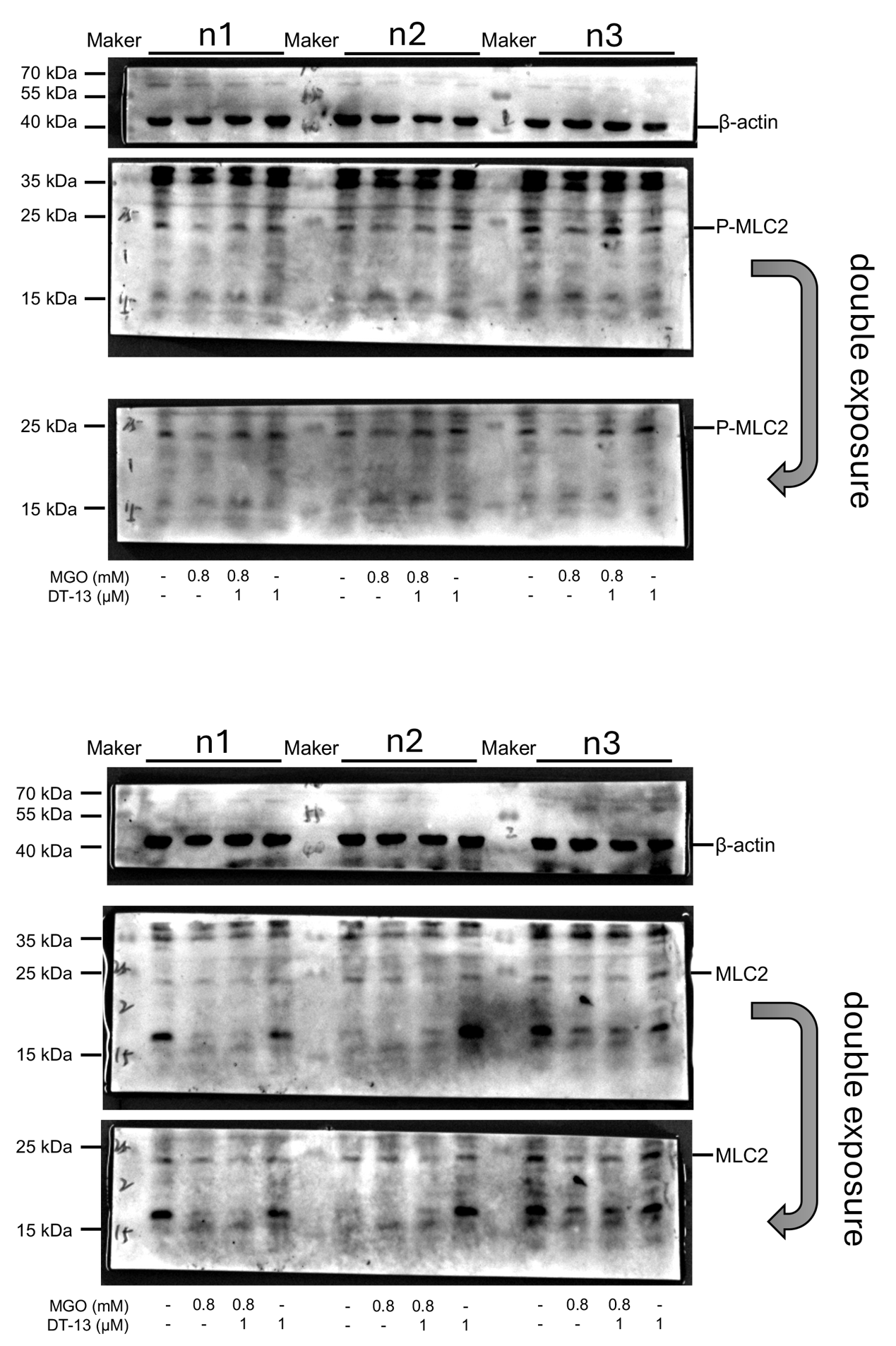


The original western blots of Figure 5E


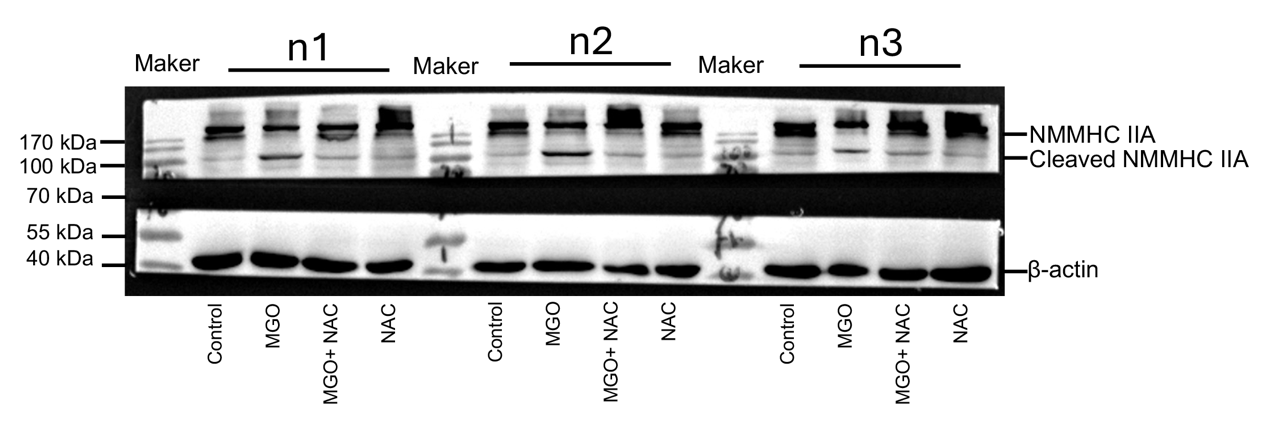


The original western blots of Figure 6E (left)


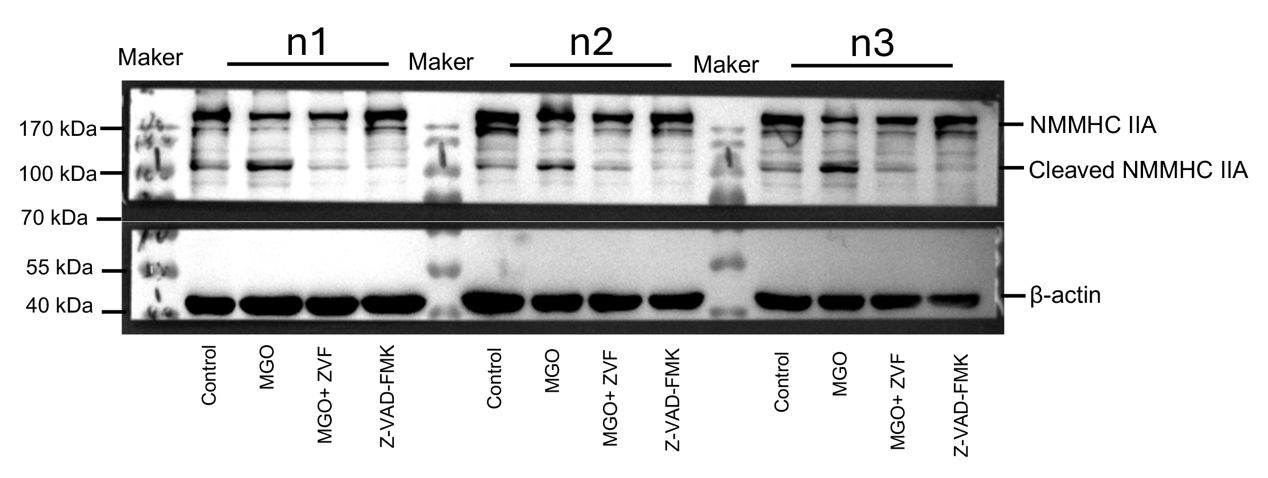


The original western blots of Figure 6E (middle)


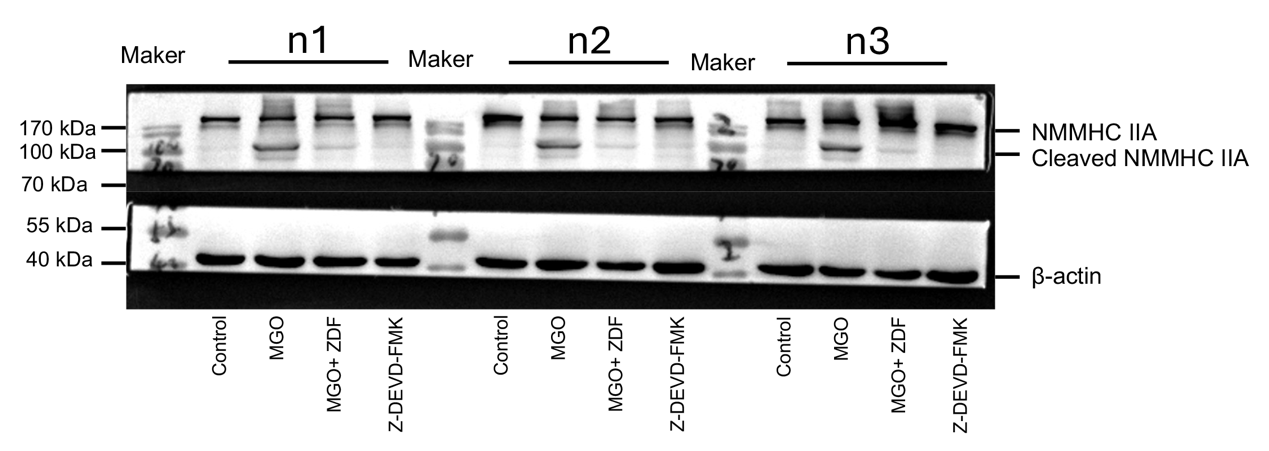


The original western blots of Figure 6E (right)


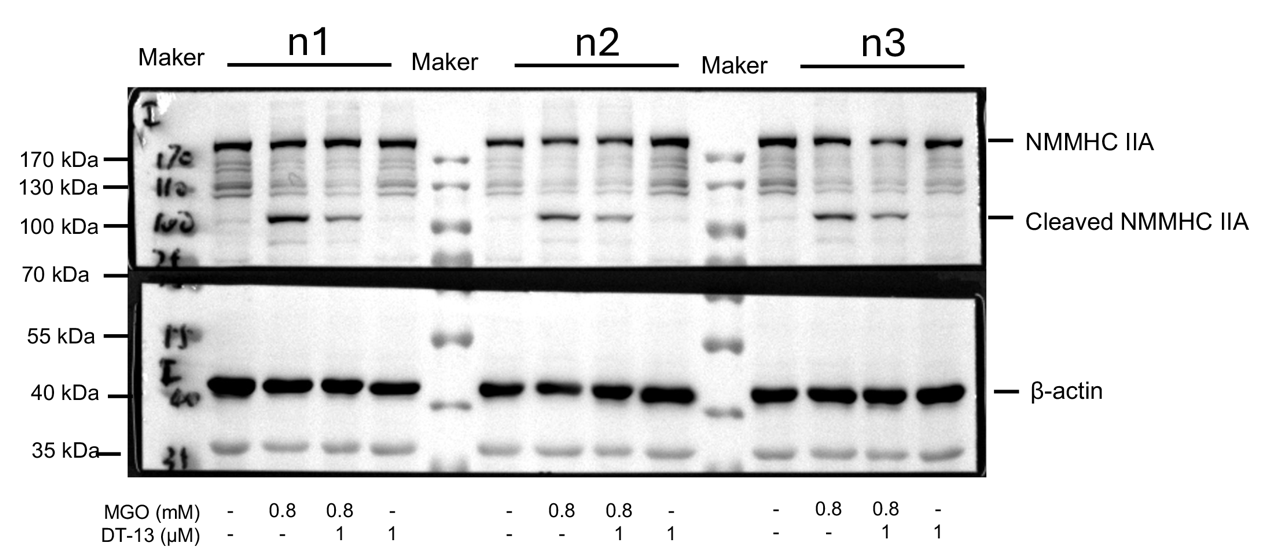


The original western blots of Figure 6F


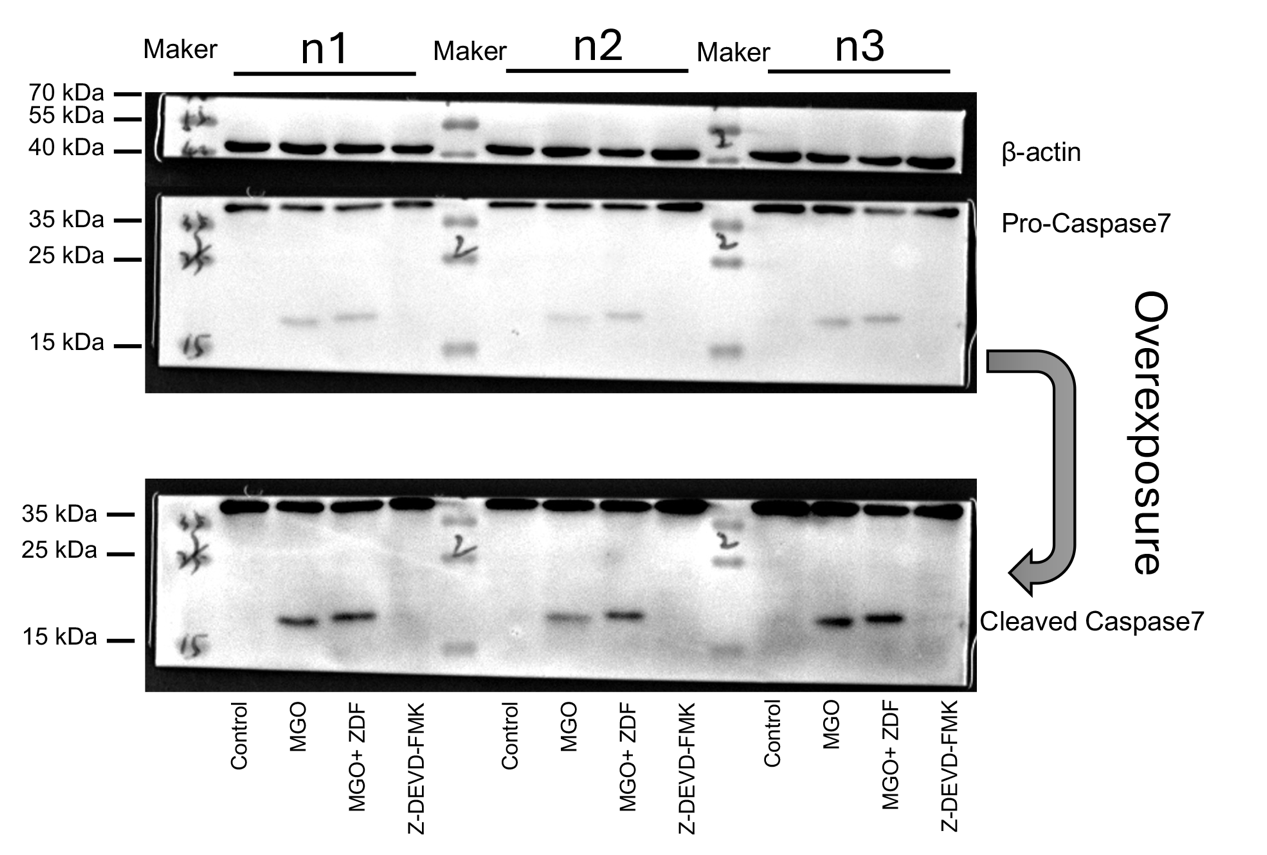


The original western blots of Supplementary Figure S1A


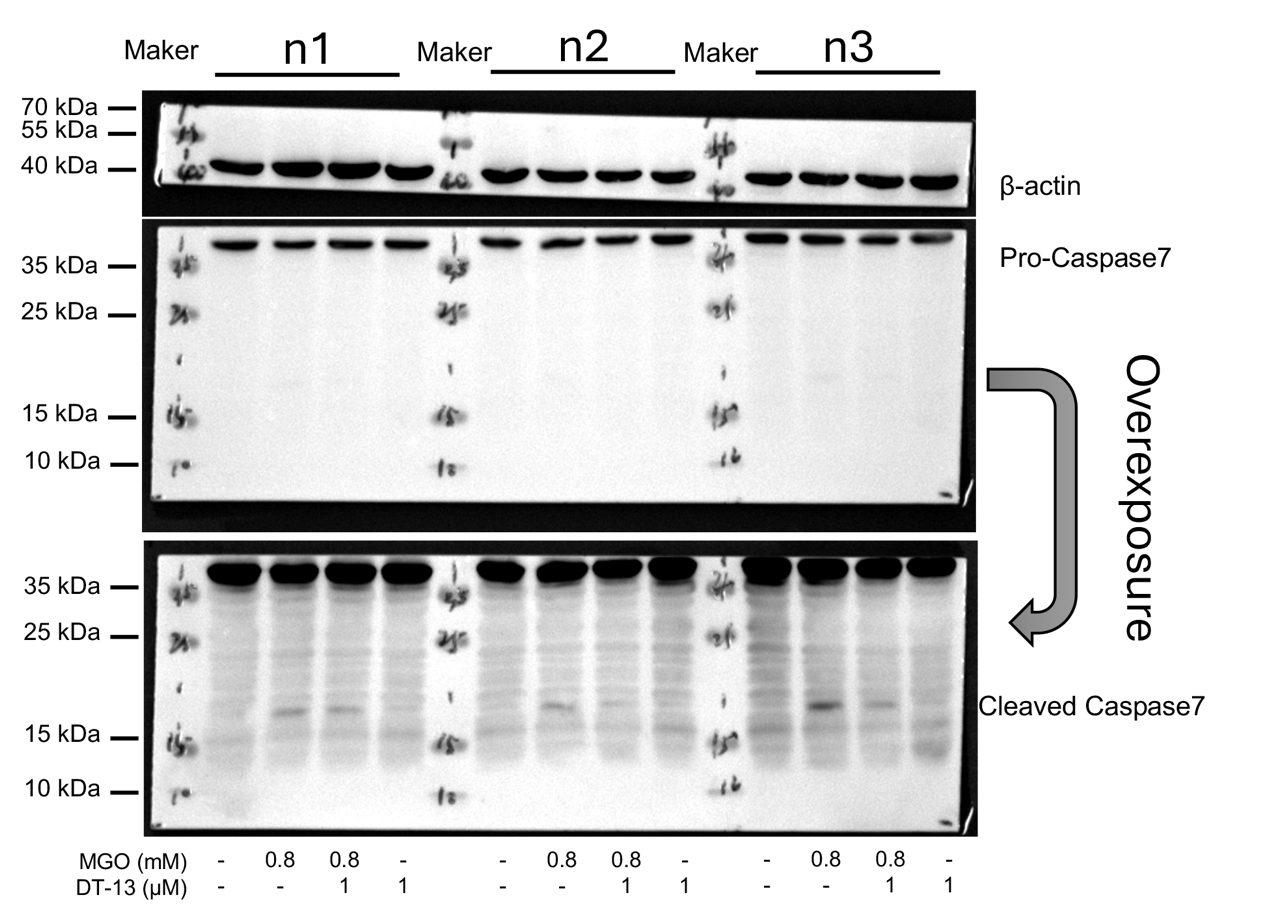


The original western blots of Supplementary Figure S1B

1. **The positive and negative control of Western blotting**


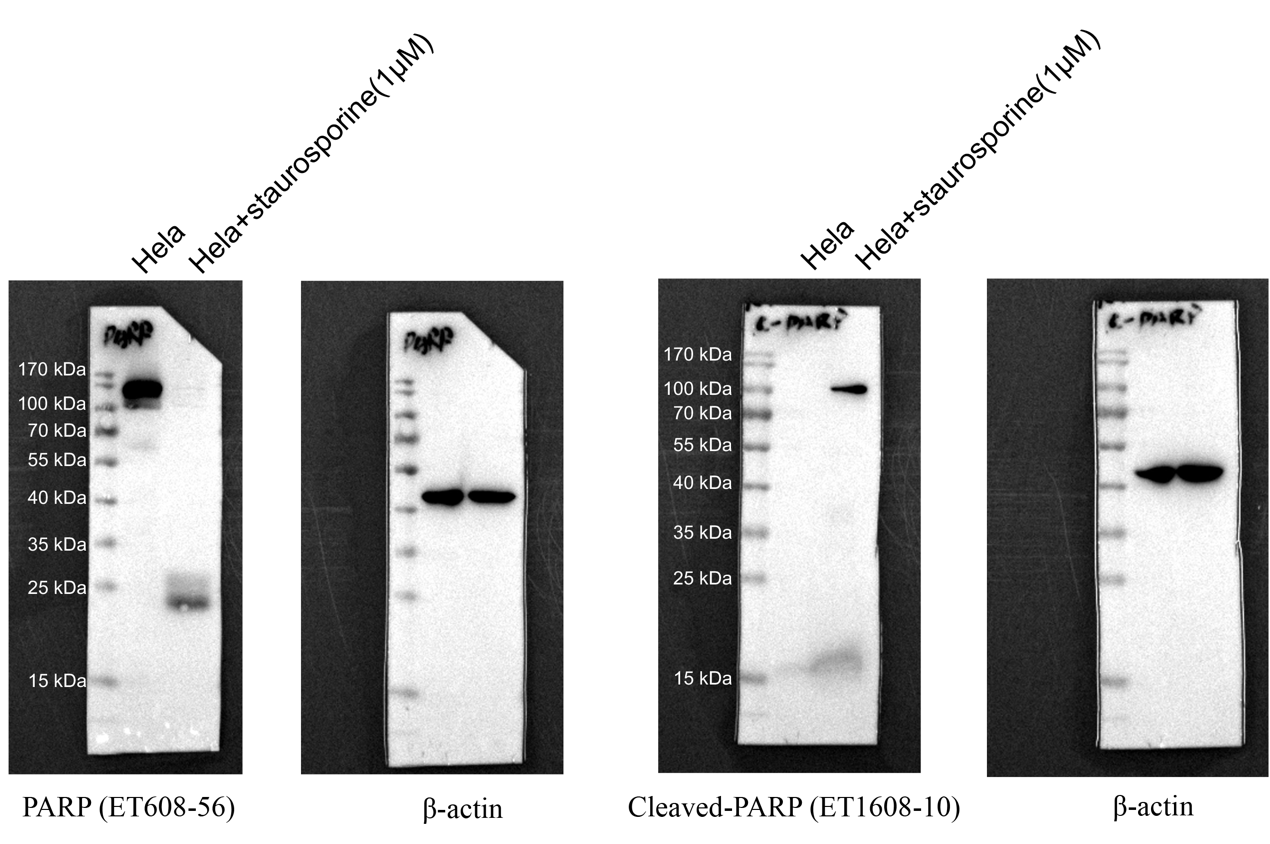


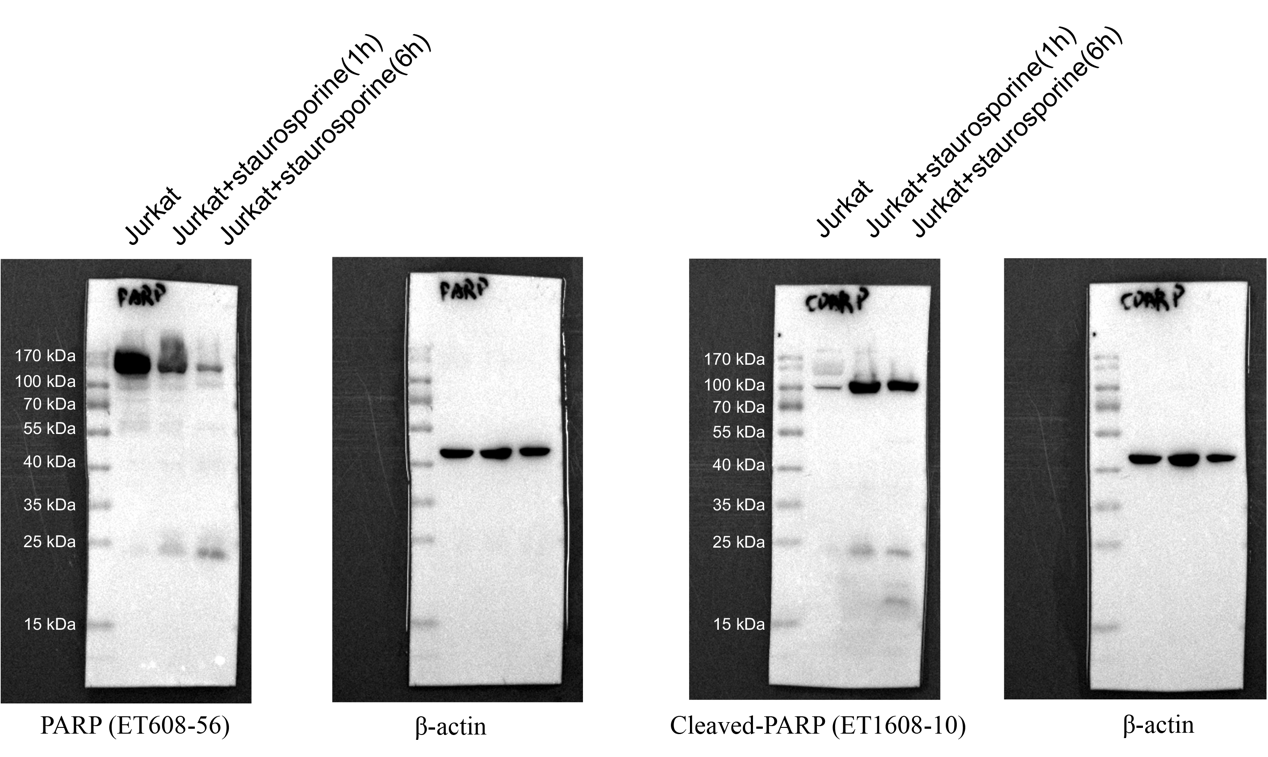


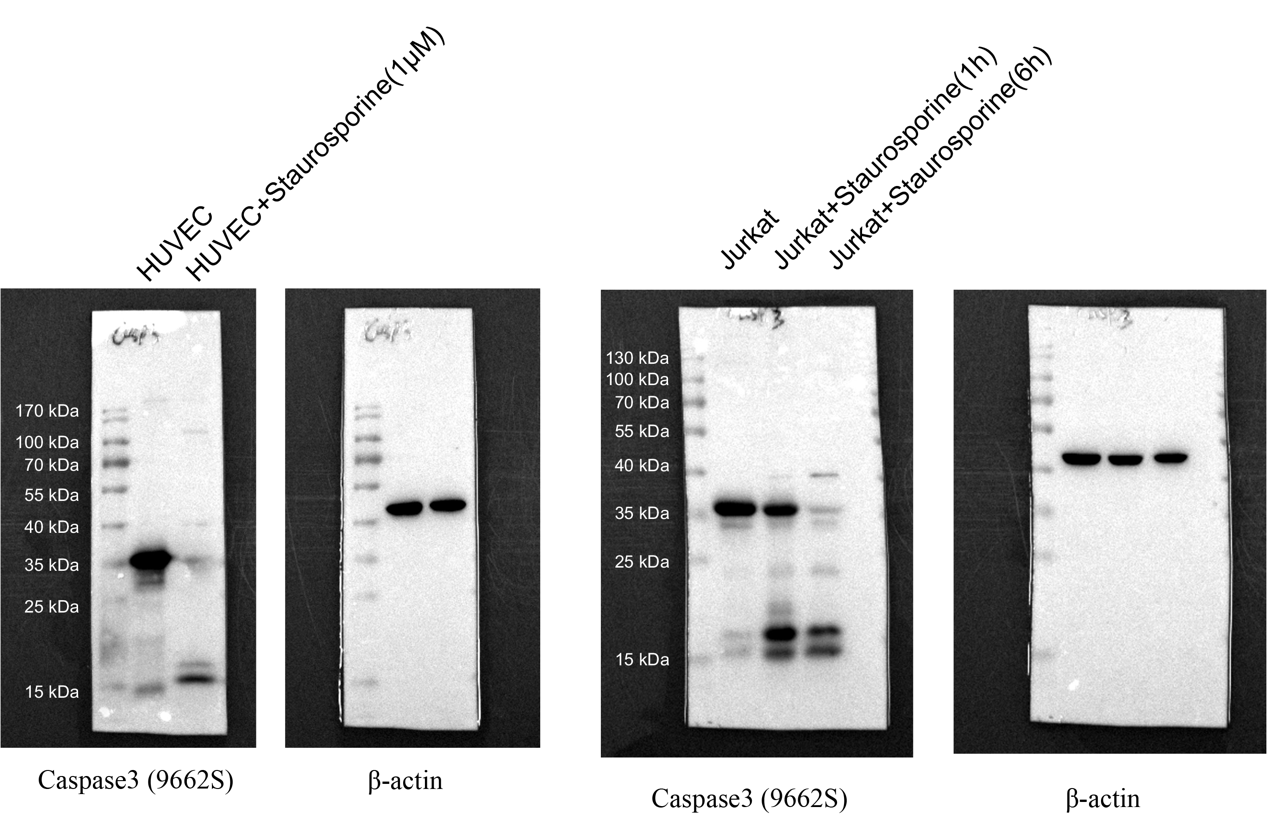


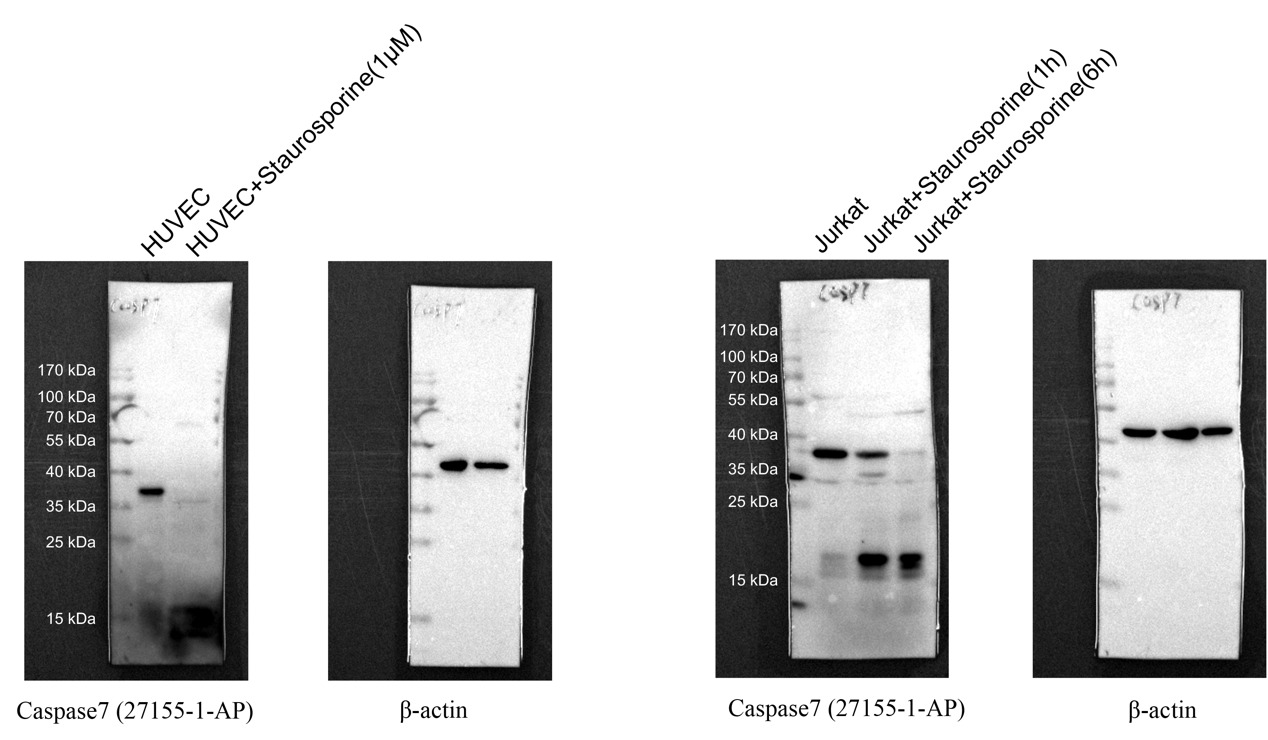


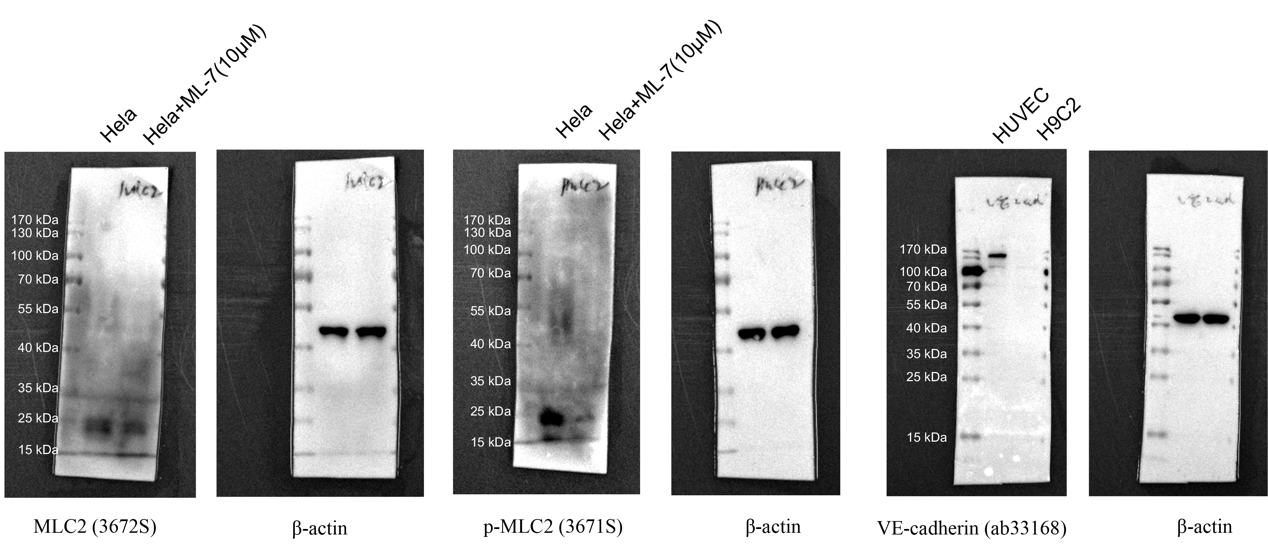


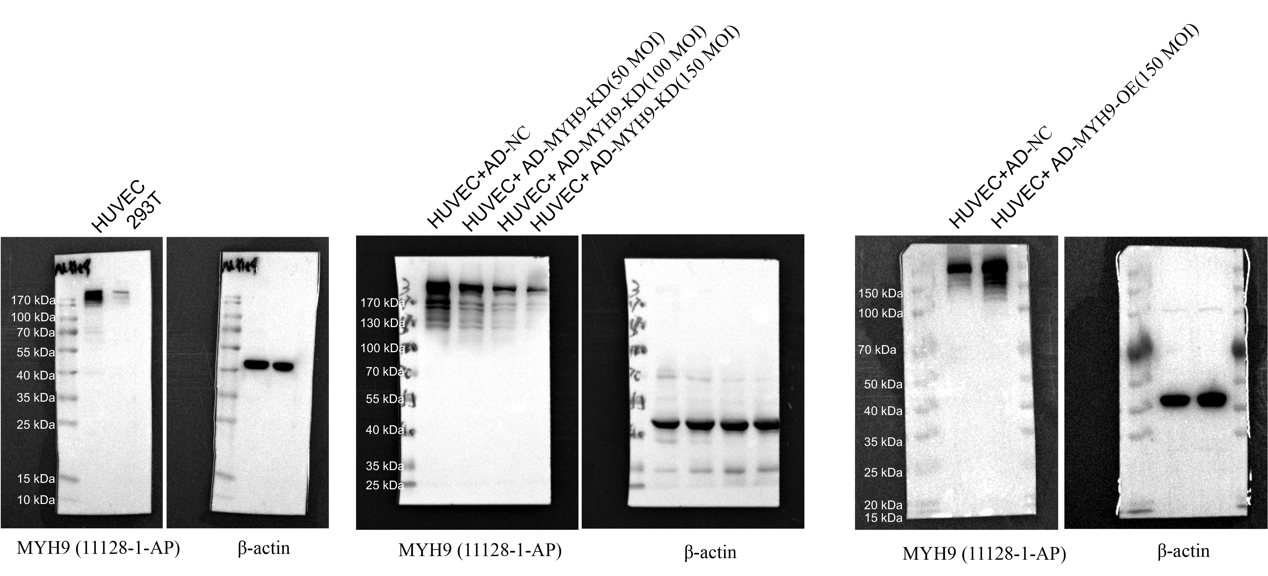


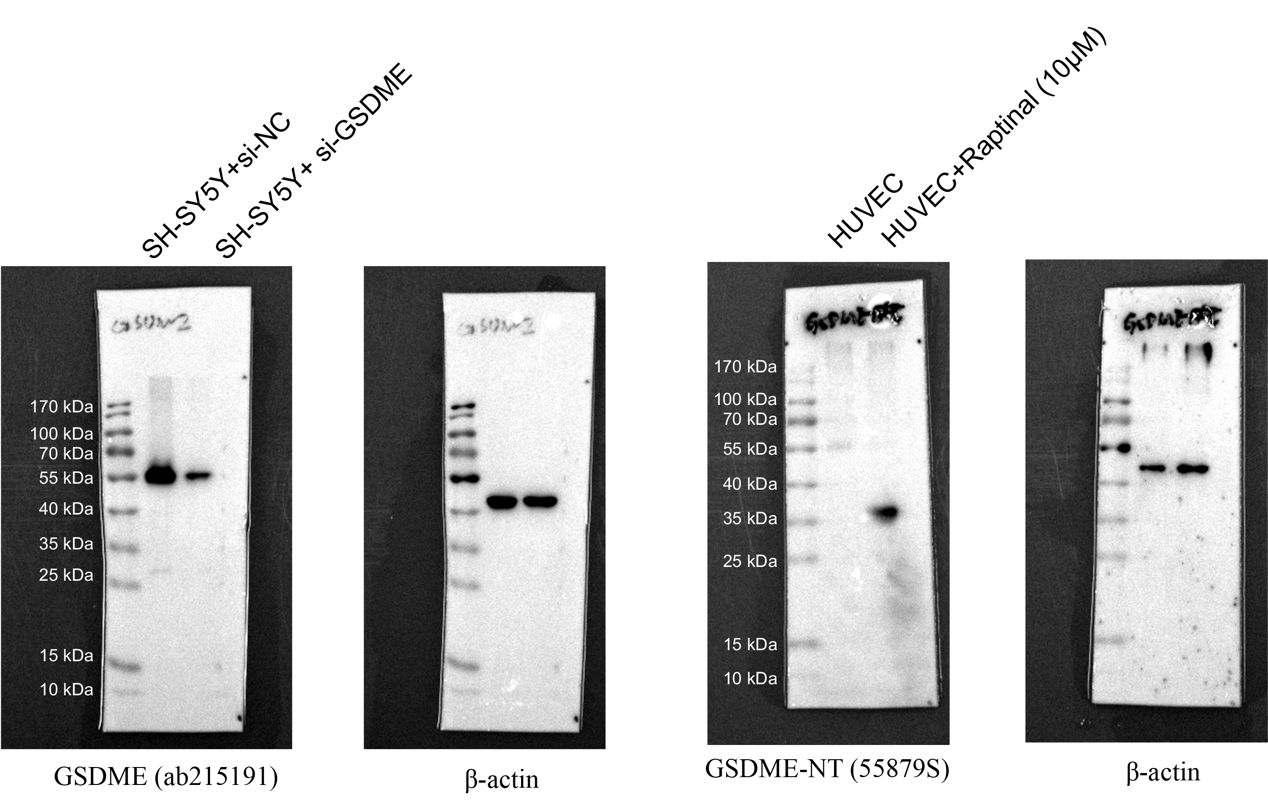


1. **The negative control of immunofluorescence**

**
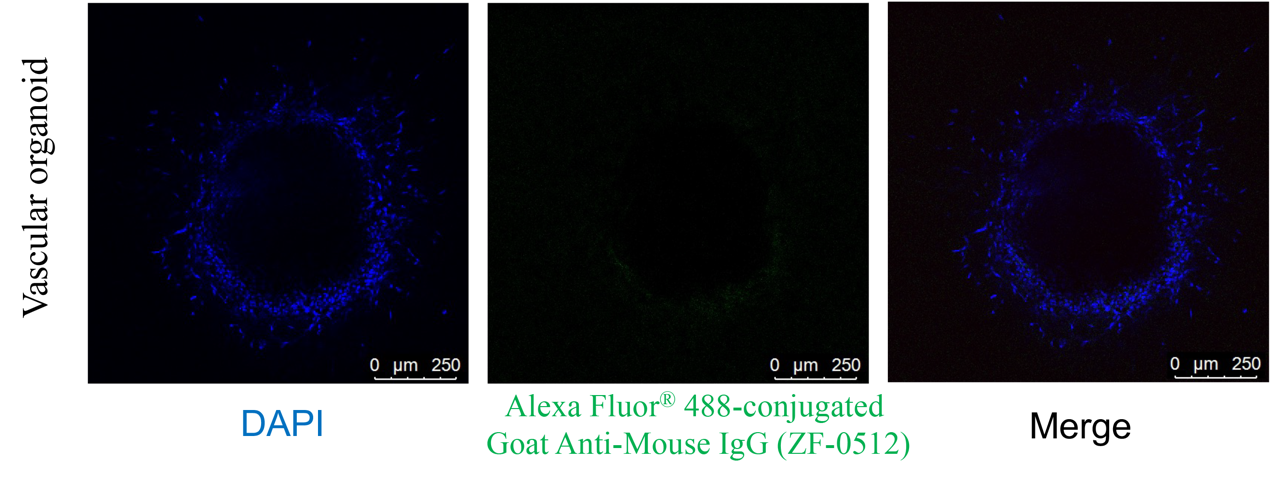
**

**
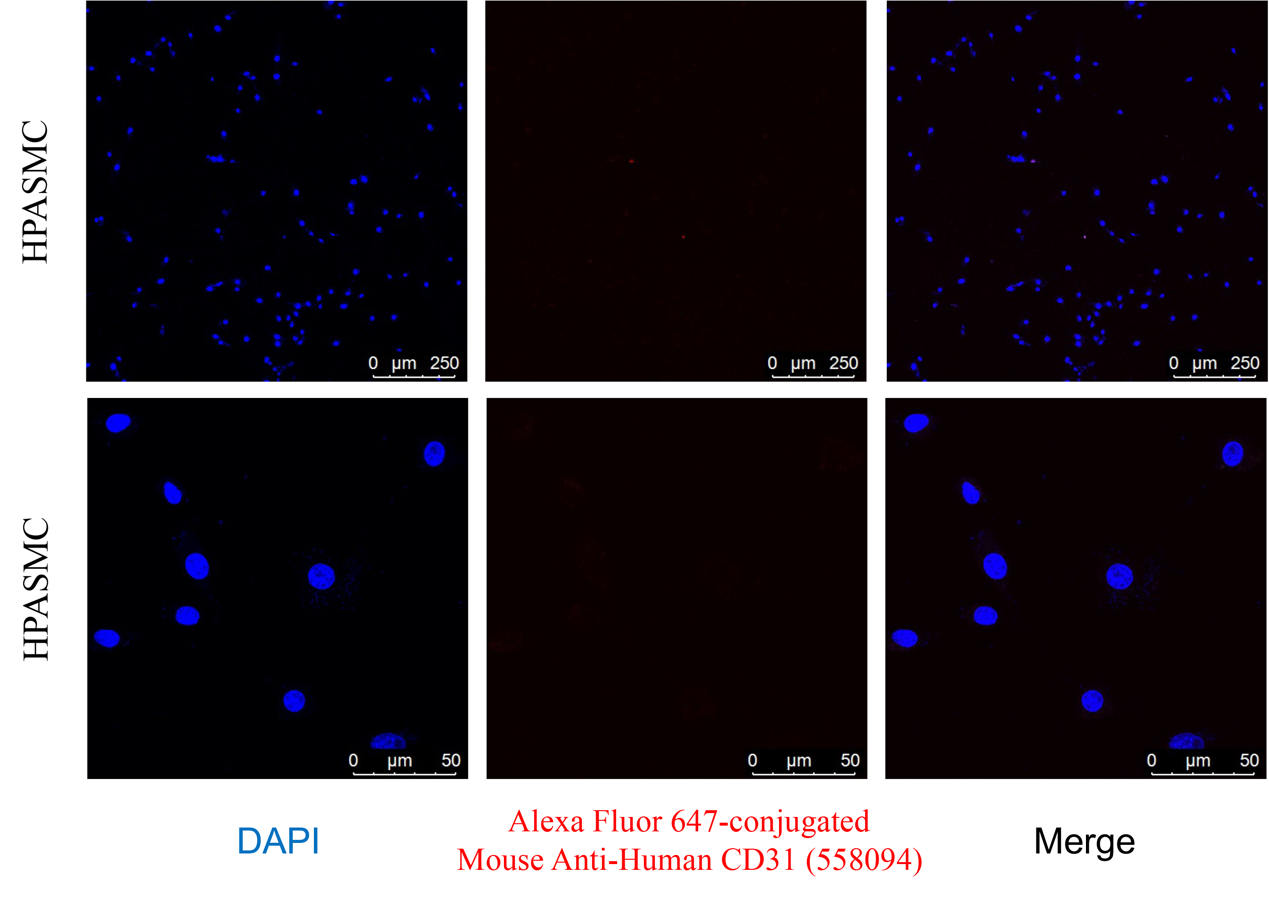
**
